# Trait dimensionality and population choice alter estimates of phenotypic dissimilarity

**DOI:** 10.1002/ece3.2780

**Published:** 2017-03-08

**Authors:** Kelly A. Carscadden, Marc W. Cadotte, Benjamin Gilbert

**Affiliations:** ^1^ Department of Ecology and Evolutionary BiologyUniversity of TorontoTorontoONCanada; ^2^ Department of Biological SciencesUniversity of Toronto‐ScarboroughTorontoONCanada; ^3^ Department of Ecology and Evolutionary BiologyUniversity of Colorado BoulderBoulderCOUSA

**Keywords:** intraspecific variation, *Mimulus* (monkeyflowers), plant functional trait, sampling decisions, species similarity

## Abstract

The ecological niche is a multi‐dimensional concept including aspects of resource use, environmental tolerance, and interspecific interactions, and the degree to which niches overlap is central to many ecological questions. Plant phenotypic traits are increasingly used as surrogates of species niches, but we lack an understanding of how key sampling decisions affect our ability to capture phenotypic differences among species. Using trait data of ecologically distinct monkeyflower (*Mimulus*) congeners, we employed linear discriminant analysis to determine how (1) dimensionality (the number and type of traits) and (2) variation within species influence how well measured traits reflect phenotypic differences among species. We conducted analyses using vegetative and floral traits in different combinations of up to 13 traits and compared the performance of commonly used functional traits such as specific leaf area against other morphological traits. We tested the importance of intraspecific variation by assessing how population choice changed our ability to discriminate species. Neither using key functional traits nor sampling across plant functions and organs maximized species discrimination. When using few traits, vegetative traits performed better than combinations of vegetative and floral traits or floral traits alone. Overall, including more traits increased our ability to detect phenotypic differences among species. Population choice and the number of traits used had comparable impacts on discriminating species. We addressed methodological challenges that have undermined cross‐study comparability of trait‐based approaches. Our results emphasize the importance of sampling among‐population trait variation and suggest that a high‐dimensional approach may best capture phenotypic variation among species with distinct niches.

## 1 Introduction

Niche theory argues that phenotypic differences among species influence interspecific interactions, environmental tolerances, and resource exploitation (De León, Podos, Gardezi, Herrel, & Hendry, 2014; Eklöf et al., 2013; Givnish, 1987). In ecology, there has been a conceptual shift to trait‐based approaches that capture these phenotypic determinants of the niche (Cadotte, Carscadden, & Mirotchnick, 2011; McGill, Enquist, Weiher, & Westoby, 2006), and this trait‐based approach is increasingly used to understand community assembly, coexistence, and ecosystem function (Díaz & Cabido, 2001; Shipley, Vile, & Garnier, 2006; Silvertown, 2004).

Despite an increased interest in trait‐based approaches, methodological issues and critical assumptions may limit their general applicability. Although the number (Maire, Grenouillet, Brosse, & Villéger, 2015; Villéger, Novack‐Gottshall, & Mouillot, 2011) and identity of traits used (Harmon, Kolbe, Cheverud, & Losos, 2005; Spasojevic & Suding, 2012) may alter inferences, a wide variety of trait‐sampling approaches are used. Similarly, intraspecific variation may be a critical component of ecological patterns and processes (Bolnick et al., 2011; Violle et al., 2012), but much trait‐based work has used species‐level means or neglected trait among‐population trait variation (Albert et al., 2010). Trait‐based approaches have been advocated partly because of the generality they promise (McGill et al., 2006), but we cannot realize this potential of powerful comparison across studies and systems until we assess the consequences of different sampling strategies.

The term “trait” is variously defined. Here, a trait is any measurable morphological, behavioral, phenological, physiological, or biochemical phenotypic character. Although any of these traits may influence organismal fitness in certain environments, only traits that have been empirically or observationally linked to fitness or performance are termed “functional traits” (e.g., McGill et al., 2006). As part of our study, we examined phenotypic variation within a species pool and evaluated whether species were better differentiated by known functional traits or other phenotypic characters. We contend that many understudied traits showing variation among closely related species are likely functional in particular biotic or abiotic settings and merely lack experimentation. As is common in trait‐based studies (Cornelissen et al., 2003), we used primarily morphological traits and “soft” functional traits (e.g., plant height), more easily measurable correlates of the functional trait of interest (“hard” traits, e.g., competitive ability), and although soft and hard traits may be correlated at global scales (e.g., Díaz et al., 2004), trait relationships may vary among systems and environments (Funk & Cornwell, 2013). Therefore, we focus strictly on capturing phenotypic differences among species and emphasize that resolving tripartite trait–environment–fitness relationships for a wide range of phenotypic characters (i.e., mapping traits to niches) remains a key area for development.

Trait‐based studies often use one to 20 traits and usually rely on one of two distinct approaches to quantifying traits: “representative trait” or “high‐dimensional” approaches. The representative trait approach posits that one or few ecologically important traits determine species' success in an abiotic or biotic milieu. For example, a single trait, plant biomass, can explain over 60% of variation in competitive ability among wetland plant species (Gaudet & Keddy, 1988). Numerous studies have focused on single functional traits, such as plant height or specific leaf area (SLA), to understand competitive differences or patterns of species distribution (Falster & Westoby, 2003; Grime, 1973; Sides et al., 2014). These low‐dimensional studies use “representative traits” relevant to plant‐strategy theories. For example, the leaf economics spectrum predicts that leaf traits shaping photosynthetic investment and return determine the distribution of broad vegetative forms across climatic gradients (Wright et al., 2004). Similarly, the leaf–height–seed scheme posits that combinations of SLA, height, and seed mass characterize species' colonization abilities and responses to disturbance (Westoby, 1998).

These plant‐strategy traits are predominantly invoked in diverse assemblages but may also vary among close relatives and intraspecifically across environments. In co‐occurring willow (*Salix*) congeners, among‐species variation in several hydrological functional traits correlated with differences in habitat affinities (species' weighted average distance to the water table); for example, congeners from wetter habitats showed higher root growth rates and turgor loss points (Savage & Cavender‐Bares, 2012). Similarly, Matzek (2011) investigated 18 resource‐capture traits in pine (*Pinus*) species and found that a single trait, photosynthetic nitrogen‐use efficiency, best explained the more rapid growth of invasive compared to noninvasive pines. Within species of European forest herbs (*Anemone nemorosa* and *Milium effusum*), plant height was greater in northerly populations, suggesting that the high‐latitude populations may be more competitive (De Frenne et al., 2011).

One type of representative trait approach (exemplified by the leaf–height–seed scheme) entails sampling across “distinct” trait groups, as not all traits are equally informative. As traits are correlated, measurement of certain traits should be redundant, yielding diminishing returns as trait dimensionality increases (Laughlin, 2014). Ecologists have long grouped traits by expected similarity and function (e.g., Raunkiaer, 1934), and sampling across distinct phenotypic axes may allow us to measure fewer traits with little loss of phenotypic information; however, this intuitive sampling solution requires a rigorous test to demonstrate its broader utility.

Representative trait approaches are valued for their mechanistic link between environment and species performance (Lepš, de Bello, Lavorel, & Berman, 2006; Wright et al., 2004). They may be most appropriate when predicting species' success along a few specific niche axes or across large biogeographic gradients. Nonetheless, how well these approaches capture phenotypic variation at finer spatial scales is poorly understood, and often the “most important” niche axis is unknown (Fridley, Vandermast, Kuppinger, Manthey, & Peet, 2007).

In contrast, many ecological questions may require a high‐dimensional trait‐sampling approach. To predict whether one species might pollinate another, for example, we need to consider plant and pollinator phenological, morphological, and behavioral traits (e.g., Eklöf et al., 2013). Investigations of community assembly mechanisms (limiting similarity and habitat filtering) are also best addressed by examining species in multivariate space (Cornwell, Schwilk, & Ackerly, 2006) as multiple phenotypic traits shape an organism's interaction with its competitors and environment. Therefore, a high‐dimensional approach should better approximate the “n‐dimensional” ways in which species differ (Cornelissen et al., 2003; Pérez‐Harguindeguy et al., 2013). Using simulations, Maire et al. (2015) demonstrated that measuring more traits may better represent a community's phenotypic variation: functional diversity calculated from 10 traits (rather than five) more closely approximated the “true” community functional diversity. But measuring numerous traits on many individuals and populations quickly becomes unfeasible, and research has just begun evaluating how trait‐sampling decisions impact estimates and applications of trait data (de Bello et al., 2011).

Here, we use an observational dataset of ecologically distinct species to explicitly compare how trait dimensionality and population sampling influence estimates of species' phenotypic dissimilarity. We assess how well the traits we measure can recover phenotypic differences among species, using vegetative and floral traits in different combinations of up to 13 traits. As we aim to provide a practical sampling guide, we tease apart two key elements of dimensionality: the number of traits sampled and the types of traits included. Specifically, we evaluate the hypotheses that (1) using more traits, (2) including both vegetative and floral traits, and (3) sampling across trait groups (e.g., leaf traits, growth form traits) will best capture interspecific phenotypic differences. Lastly, we test the importance of intraspecific variation by assessing how population choice changes our understanding of phenotypic differences among species.

## 2 Materials and Methods

### 2.1 Study system and field collections

In western North America, the monkeyflower genus *Mimulus sensu lato* consists of approximately 120 species, most occurring within California. The genus includes phenotypically distinct forms, and several monkeyflower species span a considerable geographic and environmental range (Sheth, Jiménez, & Angert, 2014) and are characterized by a series of ecomorphs (Wu et al., 2008). The seven species sampled here (*Mimulus guttatus, M. leptaleus*,* M. lewisii*,* M. mephiticus, M. moschatus*,* M. primuloides*, and *M. tilingii*) are ecologically and phenotypically distinct (Table 1). Although their phenology and persistence are tightly linked to water availability (Hall & Willis, 2006; Williams & Levine, 2004), these species diverge in elevational range, microhabitat preference, and vegetative phenotype (Table 1). Furthermore, *Mimulus* species differ in pollination syndrome, and the sampled species include outcrossers and putative selfing species (Table 1).

**Table 1:**
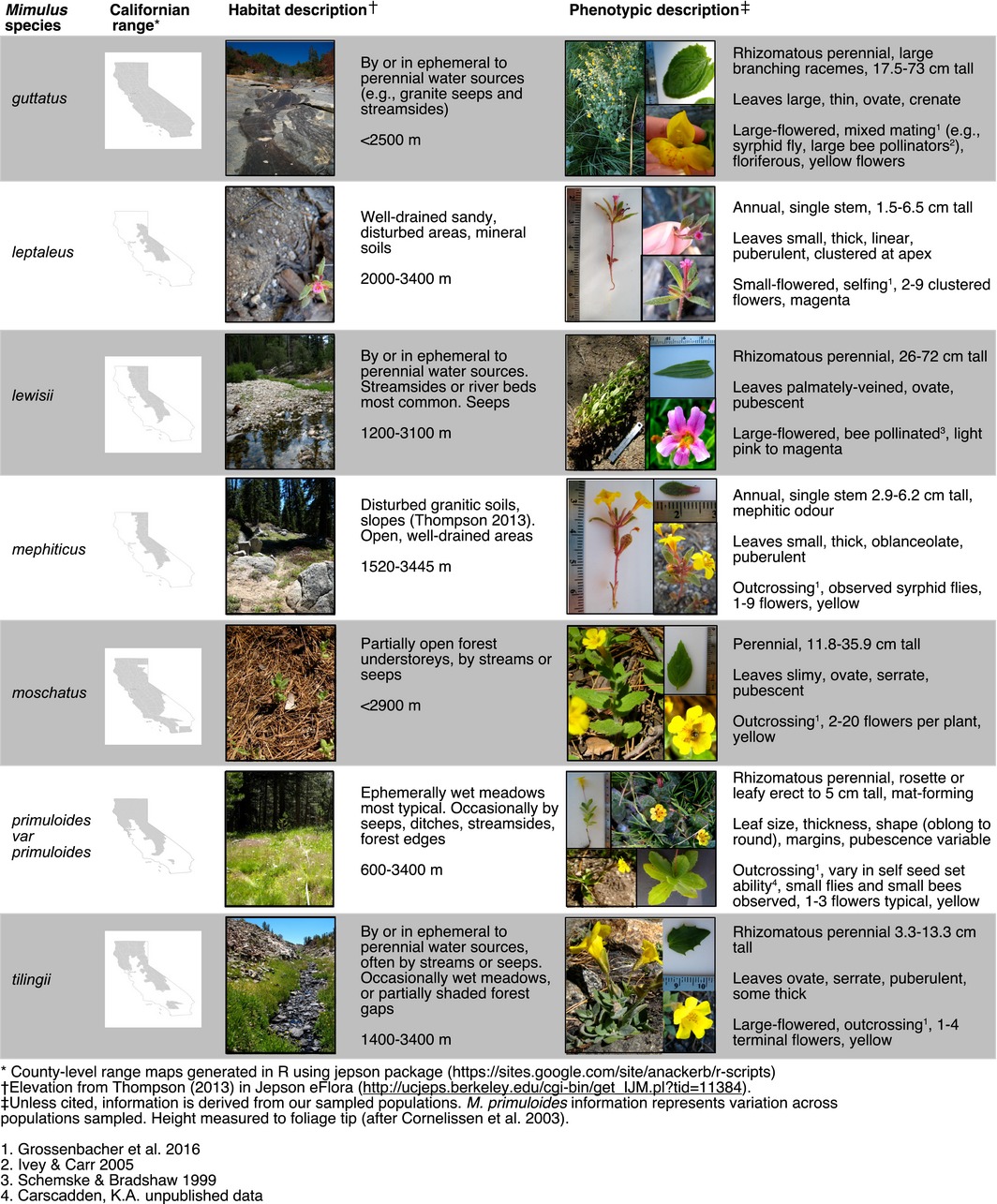
Focal *Mimulus* species: Californian geographic range, elevation range, general habitat affinities, and phenotypic descriptions

To clarify how trait dimensionality impacts measurable interspecific phenotypic differences along abiotic and biotic niche axes, we measured eight vegetative and six floral traits (Table 2) in populations of the seven focal monkeyflower species in summer 2012. We selected vegetative traits related to competitive ability, water usage, and photosynthetic capacity, and floral traits related to structural differences among species and pollen‐transfer syndromes.

**Table 2:** Measurement and grouping of vegetative and floral traits: Traits were grouped a priori by expected similarity and function

Trait dataset	Trait group	Traits	Measurement method
Vegetative	Size	Plant height	To top of vegetation
		Leaf number	Counted all leaves
	Leaf	Specific leaf area (SLA)	Fresh leaf area/dry mass^a^
		Leaf perimeter	Sum of all margins^a^
		Leaf aspect ratio	Leaf length/width^a^
		Leaf circularity	Measure of roundness^a^
	Growth form	Internode	Observed rosette or not
		Stem width	Width at basal leaf pair
Floral	Structure/investment	Flower number	Counted or estimated all
		Bud number	Counted all buds
		Pod number	Counted or estimated all
	Floral size	Corolla length	Tube length, from where corolla joins peduncle to beginning of corolla lobes
		Corolla width	Tube width, at widest point below corolla lobes
	Pollen transfer	Herkogamy	Stigma–anther separation

a Area and leaf traits measured on 2–4 leaves per individual, in ImageJ (Schneider, Rasband, & Eliceiri, 2012).

Circularity = 4π(area/perimeter^2^).

To include trait variation across environments, we sampled across a 1,866‐m elevation gradient in the Sierra Nevada Mountains of California, in Yosemite National Park and neighboring Inyo National Forest. Site selection was opportunistic, based on range maps, previous occurrence records, and local habitat descriptions. One species, *M. primuloides*, was more heavily sampled to capture among‐population variation across elevation and habitats, and populations spanned soggy high‐elevation meadows, river‐adjacent populations, forest gaps, and dry, disturbed trailsides and ditches. At a given site, we placed transects haphazardly to bisect a population along its length, and samples of flowering individuals were stratified across the transect. Individuals missing data for multiple traits were removed before analysis, and populations with fewer than nine individuals remaining were discarded (this threshold discussed below). This left seven populations of *M. primuloides*, in addition to two *M. moschatus* populations, and one population of each of the five remaining species. Leaf counts for the *M. tilingii* population are approximate. Herkogamy for several individuals in the *M. leptaleus* population was estimated to be zero; their miniscule flowers prevented nondestructive sampling of this trait in certain individuals, but herkogamy and floral size are often tightly linked (Sicard & Lenhard, 2011). The cleaned dataset had trait data for 9–18 individuals per population.

### 2.2 Statistical analysis

To determine whether the sampled traits could adequately capture phenotypic differences among species, we used linear discriminant analysis (LDA; Fisher, 1936; Venables & Ripley, 2002). LDA identifies linear combinations of variables that best model the phenotypic differences among species. With our data, it characterized the phenotype of each species and assigned individuals to species based on these discriminant “rules.” From this analysis, we assessed how the proportion of individuals correctly assigned to species varied with trait dataset, dimensionality, and combination. This approach also allowed us to determine the proportion of incorrect assignments – the species‐level information lost using different sampling approaches.

Prior to analysis, continuous traits (all but internode; Table 2) were *z*‐score‐transformed (e.g., Cornwell et al., 2006). We then created 100 balanced datasets, each time by randomly selecting a single population per each of the seven species, including nine individuals from each chosen population. LDA faces a mathematical “small sample size” problem as the number of traits approaches the number of samples (e.g., Sharma & Paliwal, 2015); hence, our sample size threshold of nine individuals per population was selected to maximize our sample size without excluding too many of our less highly sampled populations.

Within each balanced dataset, we sequentially chose a trait dataset (vegetative, floral, combined, or combined constrained as described below), the number of traits to include, and the exact combination of traits included, producing a reduced dataset for analysis (Fig. S1 for flowchart). Analyses were carried out iteratively: for each of the 100 balanced datasets, we ran through all permutations of trait datasets and numbers of traits, randomly sampling up to 100 different trait combinations per number of traits. This amounted to 1,021 unique trait combinations for each of the balanced datasets. For each trait combination, we calculated Gower's distance (Gower, 1971) using *daisy* within R package *cluster* (https://cran.r-project.org/web/packages/cluster/index.html) and used this distance matrix for all subsequent analyses. Gower's distance is commonly used in trait‐based ecological work because it accommodates different data types (e.g., binary, continuous) and permits missing values by ascribing them no weight in the distance calculation (e.g., Maire et al., 2015; Villéger et al., 2011). All analyses were conducted in R (version 3.1.2, https://www.R-project.org), and trait data and code are included in the supplement.

**Figure 1:**
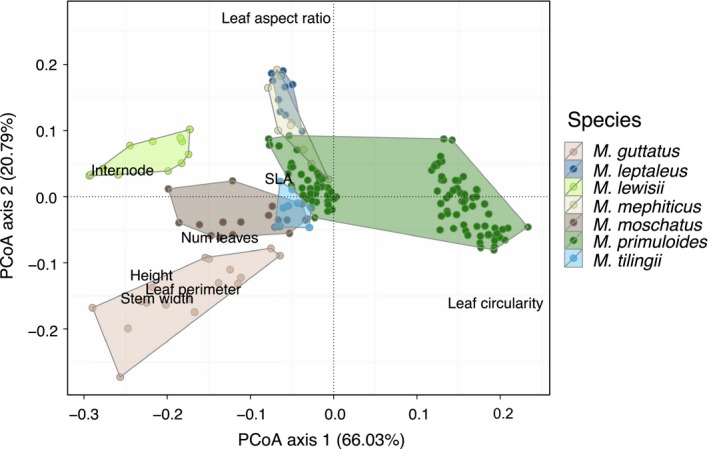
Phenotypic overlap of species in multivariate vegetative trait space. The principal coordinates analysis (PCoA) used Gower's distance on the full dataset (all populations and individuals) and all standardized vegetative traits

**Figure 2:**
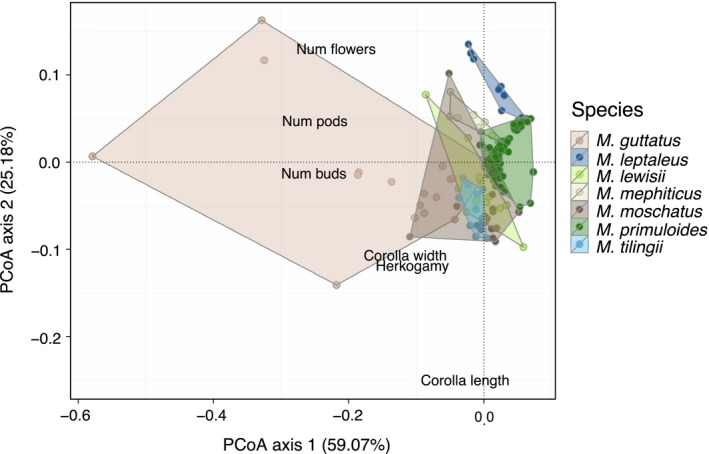
Phenotypic overlap of species in multivariate floral trait space. The principal coordinates analysis (PCoA) used Gower's distance on the full dataset (all populations and individuals) and all standardized floral traits

In a common solution to sample‐size‐based mathematical constraints of LDA (few individuals, many traits), we first used principal coordinates analysis (PCoA) on the Gower's distance matrix constructed from each selected combination of traits as a preprocessing step (Baker & Logue, 2003; Fukunaga, 1990; Sharma & Paliwal, 2015) and passed the first two major axes as input “traits” to the LDA. These first two axes should capture the vast majority of phenotypic variation: in ordinations of the full dataset, the first two PCoA axes in combination explained 86.8% of variation using vegetative traits (Figure 1), and 84.3% using floral traits (Figure 2). To further ensure that only using the first two PCoA axes from this preprocessing step did not drive the results, we conducted smaller (3 species with 12+ individuals each) parallel analyses using two and eight PCoA axes in the LDA (details in SI). With the exception of the floral‐only dataset for *M. lewisii* and *M. primuloides* population 7 (which showed greater assignment success with eight PCoA axes), the results were qualitatively very similar using two and eight PCoA axes (Figs. S2 and S3). Therefore, we report results from the larger dataset, using two PCoA axes. In previous work, “dimensionality” refers to the number of composite orthogonal phenotypic axes used (Maire et al., 2015; Villéger et al., 2011); thus, dimensionality encapsulates both the number and type of traits used to estimate phenotypic space. Our definition of dimensionality follows this concept but differs operationally. We vary the number and type of input traits, but as outlined above, our reported results are all generated using the same number of composite orthogonal trait axes (two).

To explore the impact of different ways of incorporating floral and vegetative trait data, we used four separate trait grouping approaches: vegetative traits only, floral traits only, combined traits, and combined constrained traits (explained below). In the combined traits approach, selected traits were input into a single PCoA to generate two composite “trait” axes for the subsequent LDA. This could mean that each axis contained vegetative and floral information, but it also allowed the more variable trait type to dominate. In contrast, the combined constrained traits approach used separate PCoAs such that one axis subsequently input into the LDA was constrained to be solely vegetative and the other solely floral.

We assessed whether high‐dimensional approaches provided additional phenotypic information by determining whether the proportion of individuals correctly assigned to species increased with the number of traits included. We evaluated the representative trait‐sampling approach in three ways. First, we performed LDA using single traits to determine whether species were better discriminated by single functional traits or other morphological traits (using only traits with complete field data and which were variable within subsampled species). Second, we grouped the 14 measured traits a priori into “logical” clusters thought to represent different aspects of plant function and life history. Vegetative traits were divided into plant size, leaf, and growth form traits, and floral traits comprised plant structure and investment strategies, floral size, and pollen transfer traits (Table 2). We predicted that trait combinations incorporating more of these trait groups would capture more unique phenotypic information. Third, to determine whether less strongly correlated traits would better differentiate species, we calculated the average absolute pairwise correlation within each vegetative or floral trait combination (using all individuals, populations, and species) and evaluated its average assignment success. We represent the impact of sampling decisions on correct assignment as odds ratios.

Logistically, an ideal sampling strategy entails measuring the fewest traits with minimal information loss. Therefore, we identified the best and worst four‐trait combinations (falling within the fourth or first quartiles, respectively, of assignment success across all numbers of traits included). Vegetative and floral datasets were treated separately.

## 3 Results

### 3.1 Trait dimensionality

**Figure 3:**
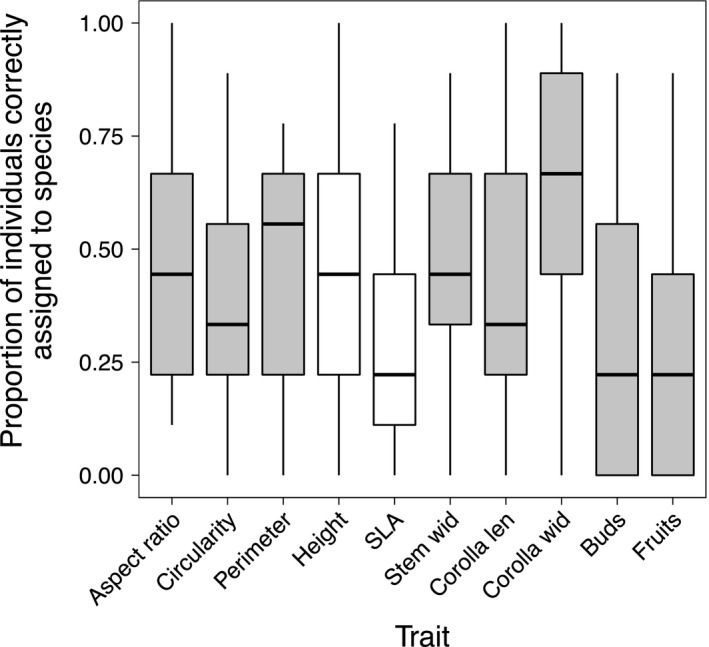
Correct assignment of individuals to species using single traits. No single trait performed best for all species, and “functional” traits such as SLA and height (white boxplots) were not noticeably better than morphological traits such as leaf aspect ratio. Only traits for which complete field data were available and which were variable within subsampled species were used in this analysis. Boxplots summarize data from all 100 runs

No single trait performed best for all species (Figure 3). Single functional traits, such as SLA and height, did not capture any more among‐species variation than did other morphological traits. Instead, different species were better distinguished by different traits (Fig. S4). For example, correct assignment of individuals to species using only SLA averaged approximately 75% for *M. moschatus* but was below 25% for several other species including *M. guttatus* (Fig. S4). Although not distinctive in several leaf traits (e.g., SLA, circularity), *M. guttatus* was best distinguished using leaf aspect ratios. Generally, *M. leptaleus* individuals were well discriminated using corolla width but not plant height. These findings suggest that to capture interspecific phenotypic differences, we need to measure multiple traits. Further, the identity of these traits may vary among assemblages.

**Figure 4:**
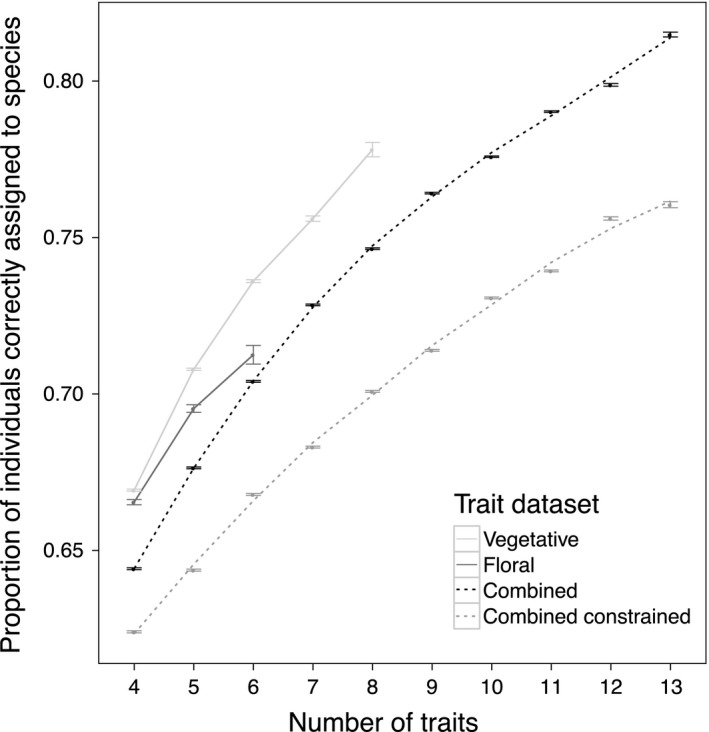
Correct assignment of individuals to species versus number of traits. Correct assignment of individuals to species increased on average as more traits were considered and varied with trait dataset used. Vegetative traits outperformed floral or combined datasets at comparable numbers of traits. The combined constrained trait dataset used separate principal coordinates analyses in linear discriminant analysis (LDA) preprocessing such that one axis subsequently input into the LDA was constrained to be solely vegetative, and the other floral. SE bars are shown

All multidimensional approaches outperformed the average single trait (41% correct assignment; Figure 3), and overall, including more traits increased correct assignment. On average, the odds of correct assignment increased about twofold over the range of numbers of traits investigated, as the proportion of correct assignment rose from 0.644 using four traits to 0.788 using 13 (Figure 4). Using few traits, combinations of vegetative traits most easily discriminated species (Figure 4). Within both vegetative and floral trait datasets, traits varied from virtually orthogonal to strongly correlated (absolute values of *r* .00–.87; Table S1), making it unlikely that differences in trait correlations drove the different performances of multidimensional vegetative and floral trait combinations. Counter to our prediction that drawing across floral and vegetative trait axes would be most informative, when few traits were used, both combined trait datasets performed below even an average of the independent vegetative and floral success (Figure 4). Using six traits, for example, the odds of correct assignment using vegetative traits were 1.4‐fold greater than with the combined constrained dataset. When more traits were used, the combined trait dataset yielded the greatest correct assignment of individuals to species (81.5% at 13 traits). However, the correct assignment rate using eight vegetative traits was similar (77.8%) and performed as well as 10 traits from the combined trait dataset. The odds of correct assignment using eight vegetative traits were 1.1 times better than using even the full combined constrained dataset (13 traits).

**Figure 5:**
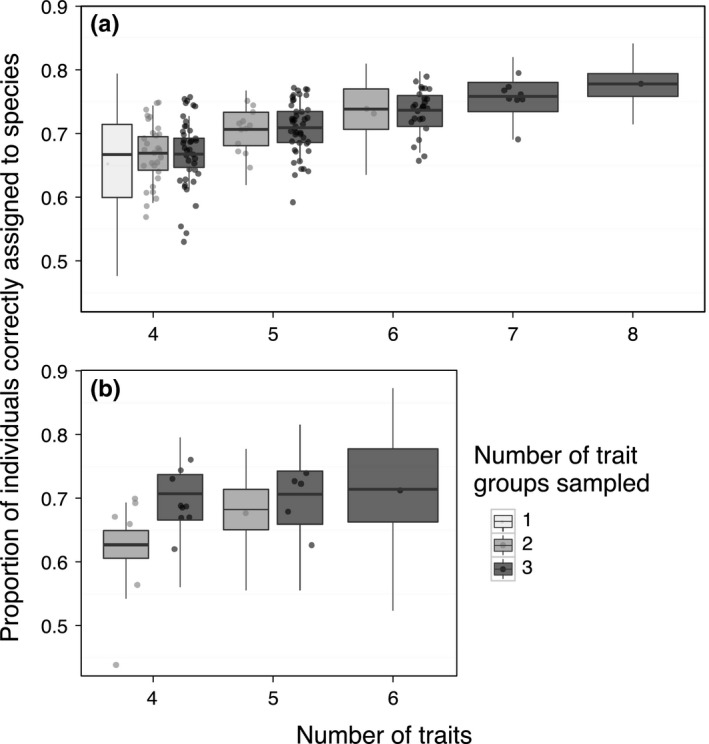
Correct assignment versus number of traits, by trait groups. The relationship between correct assignment to species and number of traits, broken down by the number of trait groups (see **Table** 2) incorporated in trait combinations, for (a) vegetative and (b) floral trait datasets. Dots indicate average correct assignment for each trait combination and are jittered to reduce overlap. Particularly for vegetative traits, sampling across trait groups did not substantially increase measurable species differences

As expected, certain trait combinations captured more interspecific phenotypic differences. Many low‐dimension trait combinations performed as well as, or better than, several higher‐dimension trait combinations. Among combinations of four traits, the odds of correct assignment using the best‐performing trait combination were greater than the least informative combination by 2.8‐ to 4.1‐fold, using vegetative and floral traits, respectively (Figure 5). Plant height and leaf aspect ratio featured in all eight of the best four‐trait vegetative combinations (Table 2 for traits). SLA appeared in 22 of the 31 worst (bottom quartile) four‐trait vegetative combinations. Leaf number, internode (rosette or not), and leaf perimeter and circularity were also common in poorly‐performing combinations. The best four‐trait floral combinations consistently contained corolla width, corolla length and herkogamy, in addition to a floral investment trait (e.g., number of flowers). In contrast, the least informative four‐trait floral combinations contained all three structural/investment traits, coupled with herkogamy or a measure of corolla size. These best and worst trait combinations were not predictable beforehand: sampling traits strategically across a greater number of “logical trait groups” (e.g., leaf traits, growth form traits; Table 2) thought a priori to capture unique phenotypic axes did not increase correct assignment (Figure 5).

**Figure 6:**
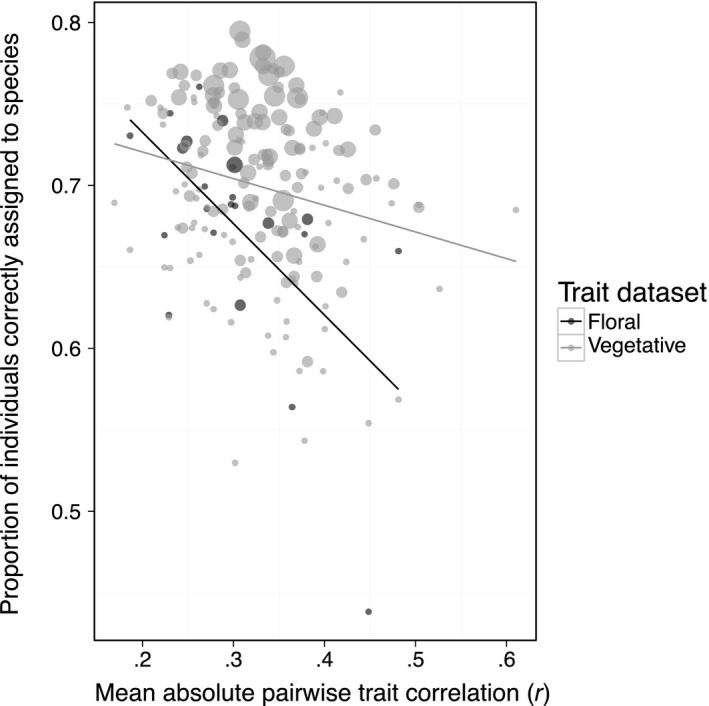
Correct assignment versus trait correlation. Correct assignment of individuals to species as a function of the average absolute pairwise correlation between traits in a trait combination, for floral and vegetative trait datasets. Points are sized by the number of traits in a combination (larger points are combinations with more traits). Correct assignment decreased as traits within a combination became more highly correlated, using floral (*y *= −0.56*x* + 0.84) and vegetative (*y* = −0.16*x *+ 0.75) trait datasets

Nonetheless, combinations of less correlated traits were more informative. A decrease in the average absolute pairwise correlation within trait combinations from 0.5 to 0.2 improved the odds of correct assignment 1.3‐ to 2.1‐fold using vegetative or floral traits, respectively (Figure 6). Although these trait datasets appear to show different trends and point spreads (Figure 6), we attribute this primarily to the larger number of vegetative combinations, spanning a wider range of numbers of traits included and assignment success.

**Figure 7:**
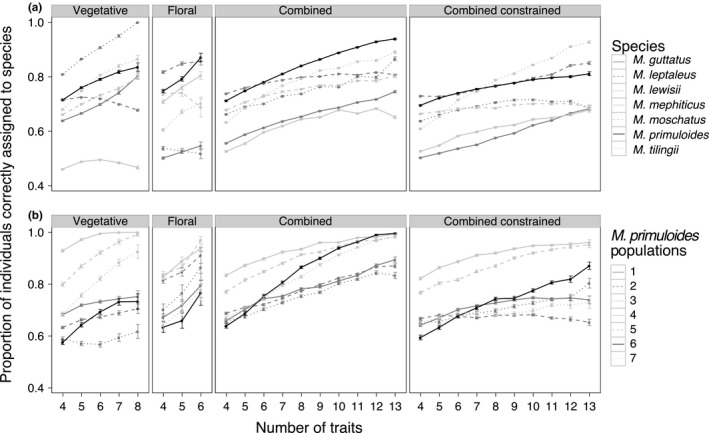
Correct assignment versus number of traits, by species and populations. The effect of the number of traits used on correct assignment of individuals to species, for all trait datasets (panels). For most species (a) and *M. primuloides* populations (b), species appear more distinct when more traits are considered. Variation among species (a) and among populations (b) in measurable species differences are of similar magnitude. Note that both (a) and (b) display how readily species were distinguished from other *Mimulus* species; only one *M. primuloides* was sampled in a given run, as part of a multispecies comparison. SE bars are shown. The combined constrained trait dataset used separate principal coordinates analyses in linear discriminant analysis (LDA) preprocessing such that one axis subsequently input into the LDA was constrained to be solely vegetative, and the other floral

Both the average assignment success and its relationship with the number of traits included varied among species. For example, using four vegetative traits, the average correct assignment was 80.9% for *M. lewisii* but only 46.2% for *M. mephiticus* (Figure 7a). Correct assignment also varied with trait dataset (panels in Figure 7a); as a case in point, *M. lewisii* was much better distinguished using vegetative rather than floral traits (80.9% vs. 53.8% success, respectively). For most species, correct assignment showed either a slight and plateauing or strong positive relationship with the number of traits, using either combined trait dataset (Figure 7a). However, using more traits did not improve correct assignment for two of seven species when a subset of traits was used (vegetative or floral), and the identity of these species differed depending on the subset used (Figure 7a). These dissimilar patterns in LDA assignment, among species and among trait datasets, are understood by examining phenotypic overlap among species in multivariate trait space. Species overlapping heavily in either vegetative (Figure 1) or floral (Figure 2) trait space were poorly discriminated using that trait dataset, even when numerous traits were considered (Figure 7a). Therefore, in speciose assemblages, multiple suites of traits would best capture species' phenotypic differences.

### 3.2 Population choice

The effects of varying the number of traits included were qualitatively similar for populations of *M. primuloides* as they were for the *Mimulus* species discussed above; however, correct assignment increased with additional floral traits for all *M. primuloides* populations (Figure 7b).

Populations differed in their phenotypic similarity with other *Mimulus* species (Figure 7b), as further evidenced by the species to which populations were most often misassigned (Fig. S5b). Population 4 was phenotypically distinctive, while when many traits were included, population 3 was most alike *M. mephiticus*, and population 2 was most alike *M. tilingii*. Population 5 of *M. primuloides* was misassigned to *M. tilingii* about 10% of the time using four traits, but hardly ever using eight traits (Fig. S5b), stressing the combined influence of trait dimensionality and population choice on species discrimination.

Population choice had a large effect on the odds of correctly discriminating species, particularly when vegetative traits were included. Within combinations of four vegetative traits, sampling the most distinct *M. primuloides* population (compared to the least distinct) increased the odds ratio by 9.6 times (Figure 7b), whereas the odds ratio increased just under fivefold between the least and most distinct species (*M. mephiticus* and *M. lewisii*, respectively; Figure 7a). Population choice also had a large impact on correct assignment compared to the effect of increasing the number of traits included: using the combined trait dataset, increasing the number of traits from 4 to 13 resulted in a 2.4‐fold boost in the odds of correct assignment (Figure 4).

## 4 Discussion

Our results demonstrate that trait and population sampling decisions have important impacts on our ability to estimate phenotypic differences among species. Using ecologically distinct congeners, we showed that high‐dimensional trait sampling estimates phenotypic differences among species better than representative traits chosen a priori. Moreover, our results show that the trait combinations that distinguish species may change not only as different species are considered, but also as different populations within species are included in an analysis. These findings have important implications for the way that trait sampling is conducted and compared across studies and for how phenotypic differences among species are interpreted.

### 4.1 Trait dimensionality

Trait dimensionality is increasingly recognized as an important issue in ecology and evolutionary biology. It can alter which mechanisms we believe are driving patterns of functional diversity (Maire et al., 2015), clarify why we detect local adaptation in some studies but not others (MacPherson, Hohenlohe, & Nuismer, 2015), and as demonstrated here, shape our perception of phenotypic differences among species.

The utility of a representative trait approach has been shown by studies comparing different trophic levels or growth forms and looking across diverse communities and environments, often at a biogeographic scale. Examining the number of traits needed to predict species interactions in different ecological networks, Eklöf et al. (2013) analyzed studies using 6–21 traits and reported that little improvement was seen beyond three traits. In these studies, 11%–100% of network structure was predictable using even a single trait, although the identity of this key trait varied among networks. Similarly, plant height is a compelling representative trait of plant competitive ability, particularly when trying to capture competitive differences among very different growth forms and along a single resource axis: light (Falster & Westoby, 2003). Lastly, leaf economics spectrum traits have successfully predicted growth and survival of diverse plant types (Poorter & Bongers, 2006) and explained variation in litter decomposition across biomes (Cornwell et al., 2008; but see Jackson, Peltzer, & Wardle, 2013 who demonstrated that within‐species variation in leaf economics spectrum traits did not explain litter decomposition).

Our study found no evidence that species differed more in “functional” traits (potentially relating to resource acquisition, competitive interactions, or plant–pollinator dynamics) than they did in other morphological traits (Figure 3). Although it has been argued that only traits with clear ecological function should be incorporated in ecological studies (e.g., Lepš et al., 2006), other traits may be equally important for several reasons: the definition of “functional trait” can be very broad and context‐dependent (McGill et al., 2006), isolating ecologically relevant traits along single environmental axes is challenging, and excluding traits becomes increasingly difficult as we consider the numerous axes forming a species' biotic and abiotic niche.

Another approach to identifying representative traits entails selecting orthogonal trait axes. For example, to understand niche variation along one important ecological spectrum (woody plant strategy), Kraft, Valencia, and Ackerly (2008) sampled “distinct” life form, leaf, wood, and seed trait axes. However in our study, vegetative trait combinations outperformed combinations of vegetative and floral traits, at a given number of traits (Figure 4). Further, the most successful combinations of few traits were not predictable beforehand based on inclusion of different trait groups (Figure 5).

Nonetheless, combinations of less highly correlated traits did detect more interspecific phenotypic differences (Figure 6). Due to trait correlations, even datasets of up to 67 traits measured on over 40 species can be condensed into about six orthogonal composite “trait” dimensions (Laughlin, 2014). Perhaps, then, the major challenge in implementing Laughlin's (2014) recommendation to sample across independent trait axes lies in identifying these orthogonal axes *before* measuring traits, as traits may be highly correlated across organs and predicted functions.

Our sampling revealed some expected and some more surprising patterns in pairwise trait correlations across species (Table S1). Among vegetative traits, plant height, stem width, and leaf perimeter showed the highest pairwise correlations (*r* = .71–.87), forming a vegetative axis of plant size. To a lesser degree, leaf number was also correlated with these size‐axis traits (*r *= .40–.52). Among floral traits, floral buds, flowers, and seed pods were most highly correlated (*r *= .58–.73), indicating phenological overlap among different stages of floral production (plants with numerous buds tended to simultaneously have numerous open flowers and maturing seed pods). Thus, these traits comprise an axis of floral production, where certain plants are generally more floriferous.

Many of the highest pairwise correlations among vegetative and floral traits were seen among these vegetative size and floral production traits (e.g., *r *= .76 between stem width and the number of floral buds; Table S1). This suggests that larger plants produce a greater absolute number of reproductive structures, consistent with work showing that larger plants even allocate relatively more (given their vegetative biomass) in reproduction as nutrient levels increase (e.g., Sugiyama & Bazzaz, 1998). Across angiosperm evolution, transitions from outcrossing to self‐fertilizing are so often accompanied by reductions in floral size and herkogamy that small flowers and low stigma–anther separation have been described as part of a “selfing syndrome” (Sicard & Lenhard, 2011), and, consequently, we had anticipated that some of our highest trait correlations might be among measures of corolla size and herkogamy. Unexpectedly, herkogamy was most highly correlated with vegetative size traits rather than floral size traits.

These relatively high correlations among vegetative and floral traits may help explain the combined constrained dataset's poor performance. The average absolute pairwise trait correlation between floral and vegetative traits is nearly identical to that within either floral or vegetative trait groups (*r* = .3). Whereas for all other datasets, the two composite “traits” used in the LDA were orthogonal (produced by a single PCoA), the separate floral and vegetative axes used in the combined constrained approach may still have contained redundant information.

Our results highlight that trait choice impacts estimates of interspecific phenotypic similarity, as the sampled species were generally more distinct along vegetative axes. In contrast, certain species, such as *M. mephiticus*, were only well distinguished using floral traits. That is, some species will be redundant along one axis but unique along others. Therefore, although including more traits increased the average phenotypic differences captured (Figure 4), if the goal is instead to ensure that phenotypic differences are adequately captured for all species, researchers may need to identify and include those trait axes that best distinguish certain suites of species. Although the floral traits appeared somewhat conserved across these tube‐flowered species, floral traits may differentiate species with greater phylogenetic scope (encompassing disk flowers of *Aster* and spikes of *Pedicularis*, for example).

In our study, correct assignment increased with trait dimensionality. This support for a high‐dimensional approach is echoed in the literature. For example, Villéger et al. (2011) assessed functional changes in marine benthos across geologic time using two to four composite orthogonal “trait” dimensions. Only the highest trait dimensionality revealed significant functional dissimilarity among assemblages. In addition, Laughlin (2014) analyzed trait datasets from six different systems and consistently found that including more traits improved predictions of community composition (by better resolving phenotypic differences among species). This positive relationship began to plateau after four to eight traits in Laughlin's (2014) study, unlike in our work. We found that additional traits revealed further interspecific phenotypic differences even when considering many more traits than commonly used in trait‐based studies that include intraspecific variation. This suggests that much of the trait literature may be underestimating phenotypic variation among species.

As support for both representative and high‐dimensional approaches can be found in the literature, we propose that (1) geographic scale and (2) question scope may delineate when each approach is preferable. The leaf–height–seed scheme, a key example of the representative approach, was designed for comparisons at a global, rather than regional or community, scale (Westoby, 1998). Similarly, at local scales, trait relationships within the leaf economics spectrum may be weaker and context‐dependent, influenced by environment, historical biogeography, and a reduction in trait variation (Funk & Cornwell, 2013; Wright et al., 2004). Within herbaceous systems such as ours, seasonality limits leaf life span, reducing this trait's variation and responsiveness to other leaf economics traits (Funk & Cornwell, 2013). That is, herbaceous plants that invest in thicker leaves may not see a corresponding increase in leaf longevity, possibly reducing the usefulness of this suite of traits for many communities. A high‐dimensional approach may be most appropriate at regional and community scales, where trait diversity is shaped by a series of environmental “filters,” each potentially acting on different traits (Lavorel & Garnier, 2002).

Certain questions may be best addressed using a high‐dimensional approach. In a French Alpine grassland study, models including abiotic variables and just two of five measured plant traits best predicted ecosystem properties such as green biomass and soil carbon (Lavorel et al., 2011). However, because these different ecosystem properties invoked nonoverlapping sets of traits, understanding ecosystem multifunctionality would require more traits. Similarly, Kraft, Godoy, and Levine (2015) found that no single functional trait explained observed patterns of plant coexistence; instead, stabilizing niche differences were only discernable when multiple functional, structural, and phenological traits were considered, as plants may partition their environments along numerous axes. Future research should continue to clarify when higher trait dimensionality is necessary.

High‐dimensional trait sampling is compatible with the three major goals of functional ecology, distilled by McGill et al. (2006): elucidating mechanisms, achieving generality across systems, and moving toward predictive functional ecology. With a high‐dimensional approach, post hoc analysis may help identify trait–environment relationships and candidate traits for subsequent experimentation. Although representative strategies have greatly advanced functional ecology by providing useful comparisons at the biogeographic scale, high‐dimensional approaches need not preclude some degree of generality. Westoby and Wright (2006) have expanded the leaf–height–seed scheme with additional traits (e.g., root:shoot ratios, growth strategies), and reproductive traits could be incorporated. Lastly, Lavorel and Garnier (2002) proposed dividing traits into “response” and “effect” traits, to predict how community functioning will be impacted as traits respond to environmental change. Fitting additional traits into this framework is worthwhile, as environmental change may occur along numerous axes and we are evermore interested in multiple ecosystem functions.

### 4.2 Quantifying phenotypic dissimilarity

Existing theory makes contrasting predictions regarding phenotypic dissimilarity of co‐occurring species. Phenotypes may diverge to reduce niche overlap and competition (limiting similarity; MacArthur & Levins, 1967). Alternatively, fitness‐related traits may converge, reducing competitive asymmetries and allowing coexistence (Chesson, 2000). Trait convergence may also result from environmental “filters” limiting the range of permissible phenotypes (Keddy, 1992).

In our study, certain species were less well discriminated than others, depending on the trait dataset used. Similarly, Harmon et al. (2005), studying *Anolis* lizard radiations, noted that specialist species converged along certain morphological axes but diverged along others. Here, *M. mephiticus* and *M. leptaleus* were sampled at the same dry, disturbed site, and *M. guttatus* and *M. moschatus* co‐occurred in a wet meadow. Perhaps due to this “harsher” shared environment, *M. mephiticus* and *M. leptaleus* had similar vegetative trait values (Figure 1). However, they had more distinctive floral traits (Figure 2), consistent with a macroecological study showing greater floral divergence in sympatric sister species in *Mimulus* (Grossenbacher & Whittall, 2011). We observed the opposite pattern of trait convergence in the *M. guttatus*–*M. moschatus* pair, perhaps due to less restrictive environmental conditions but a limited pollinator pool. Indeed, other studies have demonstrated that these two contrasting processes may operate simultaneously and that their effects may vary across traits (Cornwell & Ackerly, 2009; Kraft et al., 2008).

Studies are increasingly characterizing intraspecific variation to better understand ecological phenomena, from trophic cascades to community assembly to range shifts (Angert, Sheth, & Paul, 2011; Jung, Violle, Mondy, Hoffmann, & Muller, 2010; Post, Palkovacs, Schielke, & Dodson, 2008). In our study, different populations of a single species varied greatly in their phenotypic similarity with other species. This among‐population variation poses a challenge for trait‐based studies. At macroecological scales, it means that sampling multiple populations would most accurately depict overall similarity among species. At local scales, locally sampled trait data, rather than species means, should better represent the potentially unique confluence of genes and environment found at a site (Carmona, Rota, Azcárate, & Peco, 2015). These consequences of intraspecific variation imply that the most appropriate traits for characterizing species' phenotypes may differ among studies, even when the same species are sampled, and suggest that ideal trait combinations may vary across space.

### 4.3 Future directions

Our use of readily measurable traits is both a strength and a limitation, pointing to interesting research avenues. It allowed us to sample a relatively large number of traits across different plant organs and made possible our comparison of contrasting trait‐sampling approaches. Our study demonstrated that using more and different types of traits better captured overall phenotypic dissimilarity; however, detailed study of trait–fitness relationships across heterogeneous environments would be needed to extend this approach to understanding niche differences. In other words, to determine whether the high‐dimensional phenotypic differences we observed among species reflect differentiation across numerous niche axes (and analogously, to determine whether phenotypically similar species are functionally redundant), studies clarifying the ecological significance of a broader suite of traits and trait combinations are required. Then, analyses such as ours could profitably explore weighting traits by their correlation with environmental gradients or fitness.

In conclusion, many ecological questions require understanding species' phenotypic differences. However, despite the mounting number of trait‐based studies, our capacity to make robust conclusions and cross‐study comparisons has been plagued by a lack of consensus when it comes to sampling. Faced with measuring many traits or investing time divining the best trait combinations, one might ask: “Why traits?” Although phylogenies can, in some cases, represent phenotypic and ecological differences among species (Flynn, Mirotchnick, Jain, Palmer, & Naeem, 2011; Gravel et al., 2012), phenotypic traits propose a mechanism. For example, traits determine whether and how two organisms might interact (Eklöf et al., 2013). Our study focuses attention on methodological decisions and sampling recommendations to propel this field forward.

## Author Contributions

K.A.C. designed the study, collected and analyzed the data, and wrote the manuscript with discussion and advice from B.G. and M.W.C.
