# Supplementary material for: Trait dimensionality and population choice alter estimates of phenotypic dissimilarity
Source: Ecol Evol. 2017 Mar 8;7(7):2273–85. doi: 10.1002/ece3.2780 (PMC5383497; doi:10.1002/ece3.2780)

Supporting Information to the paper
Carscadden, K.A. et al. Trait dimensionality alters estimates of phenotypic dissimilarity. *Ecology and Evolution*.

Fig. S1. Flowchart of data subsetting. The main trajectory illustrates one sample outcome; the repeated analyses performed at each level are indicated to the left. Further details on the analyses are found in the main text.

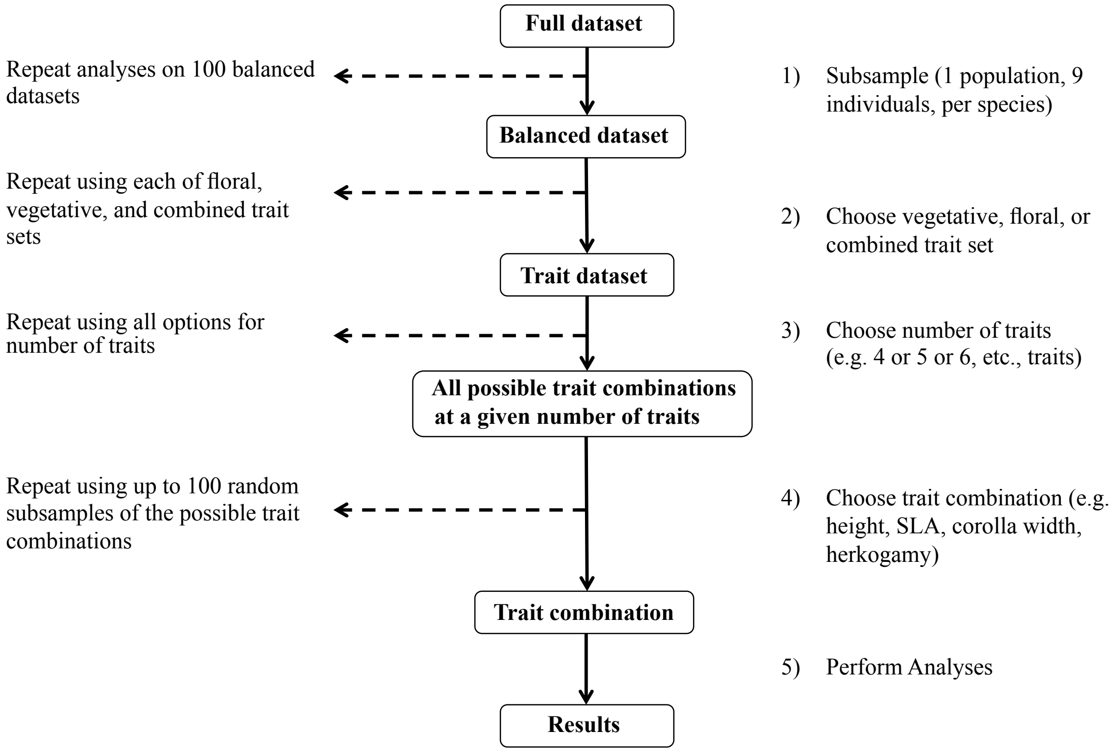

Fig. S2. Correct assignment vs. number of traits, 2 vs. 8 PCoA axes, by species and populations. Testing the effect of using different numbers of Principal Coordinates Analysis (PCoA) axes in the Linear Discriminant Analysis using three of the more highly sampled species, which had a minimum of 12 individuals each. Correct assignment is shown as a function of the number of traits across species (a) and *M. primuloides* populations (b). Panels are trait datasets. In both (a) and (b), solid lines indicate the use of 2 PCoA axes, and dashed lines denote the use of 8 PCoA axes. Results were qualitatively very similar; thus, results from the full dataset using 2 PCoA axes are reported in the main text.

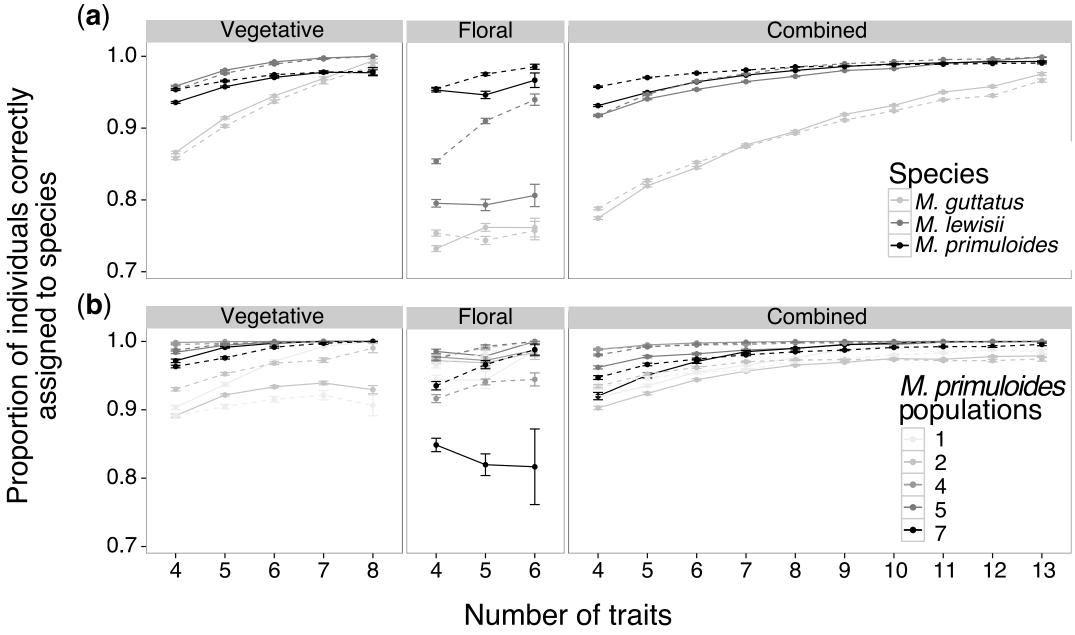

Fig. S3. Correct assignment vs. number of traits, 2 vs. 8 PCoA axes. Testing the effect of using different numbers of Principal Coordinates Analysis (PCoA) axes in the Linear Discriminant Analysis using three of the more highly sampled species, which had a minimum of 12 individuals each. Correct assignment is shown as a function of the number of traits, averaged across species. Solid lines indicate the use of 2 PCoA axes, and dashed lines denote the use of 8 PCoA axes. Trait datasets are indicated by line color. Results were qualitatively very similar; thus, results from the full dataset using 2 PCoA axes are reported in the main text.

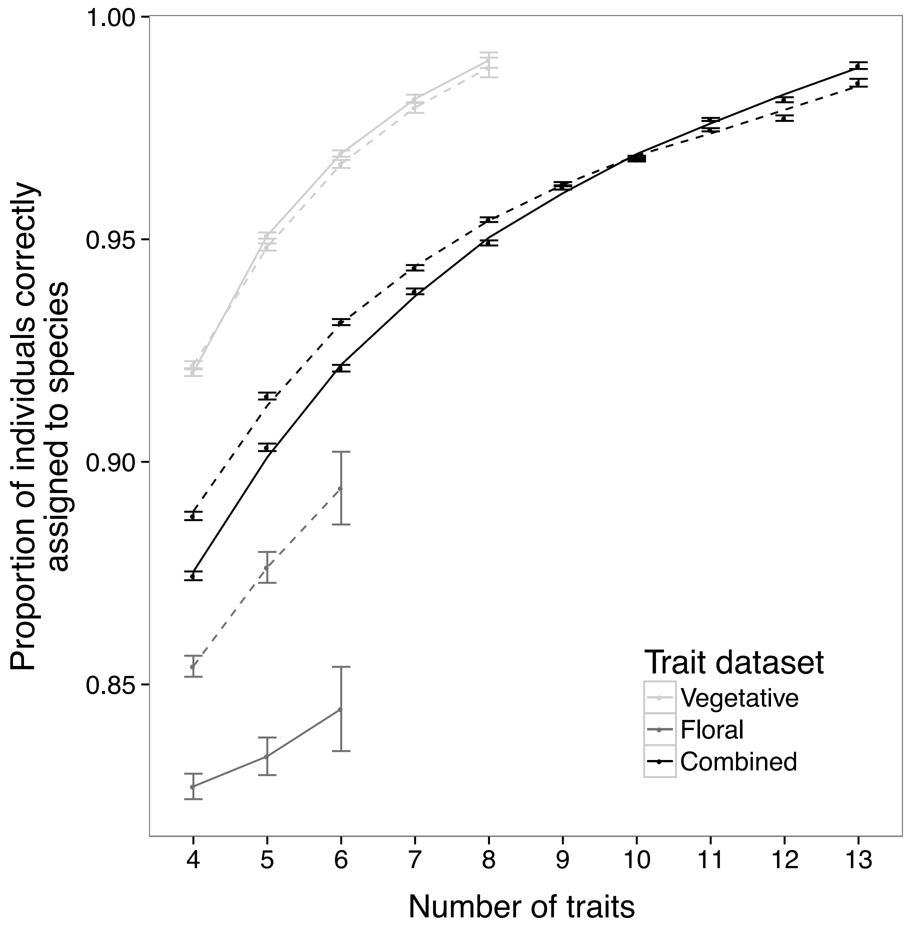

Fig. S4. Correct assignment of individuals to species using single traits, showing species means. No single trait performed best for all species, and 'functional' traits like SLA and height were not noticeably better than morphological traits like leaf aspect ratio. Only traits for which complete field data were available and which were variable within subsampled species were used in this analysis. Boxplots summarize data from all 100 runs, and symbols indicate average correct assignment for each species, for each trait.

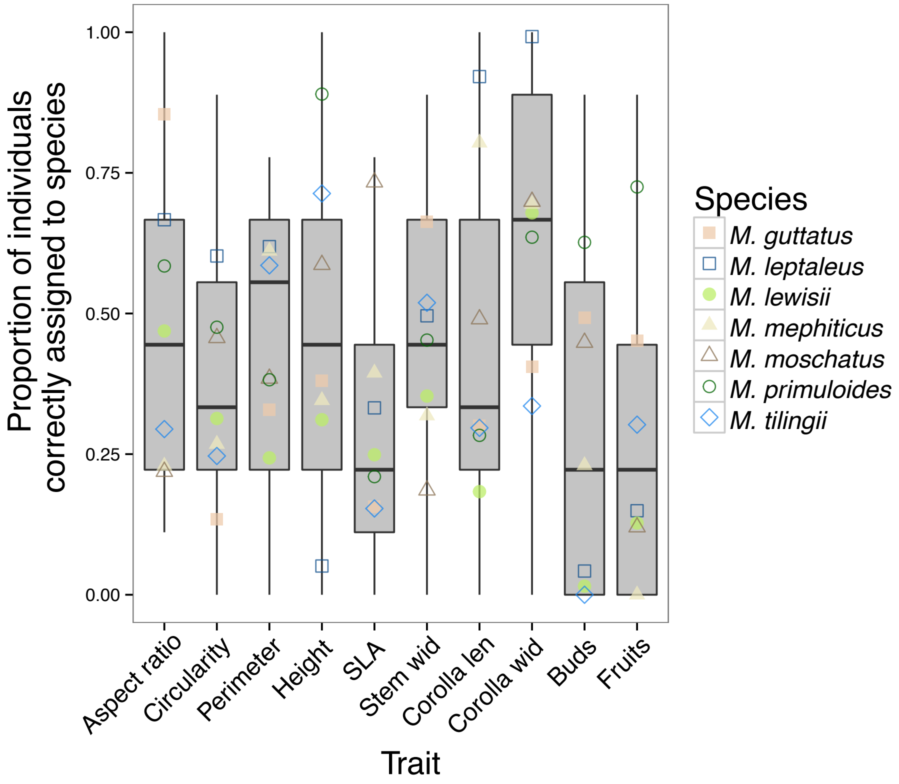

Fig. S5. Misassignment of individuals to species vs. number of traits, by species and populations. Species to which individuals were misassigned, as a function of the number of traits, using the vegetative trait dataset. Panels are species (a), or *M. primuloides* populations (b).

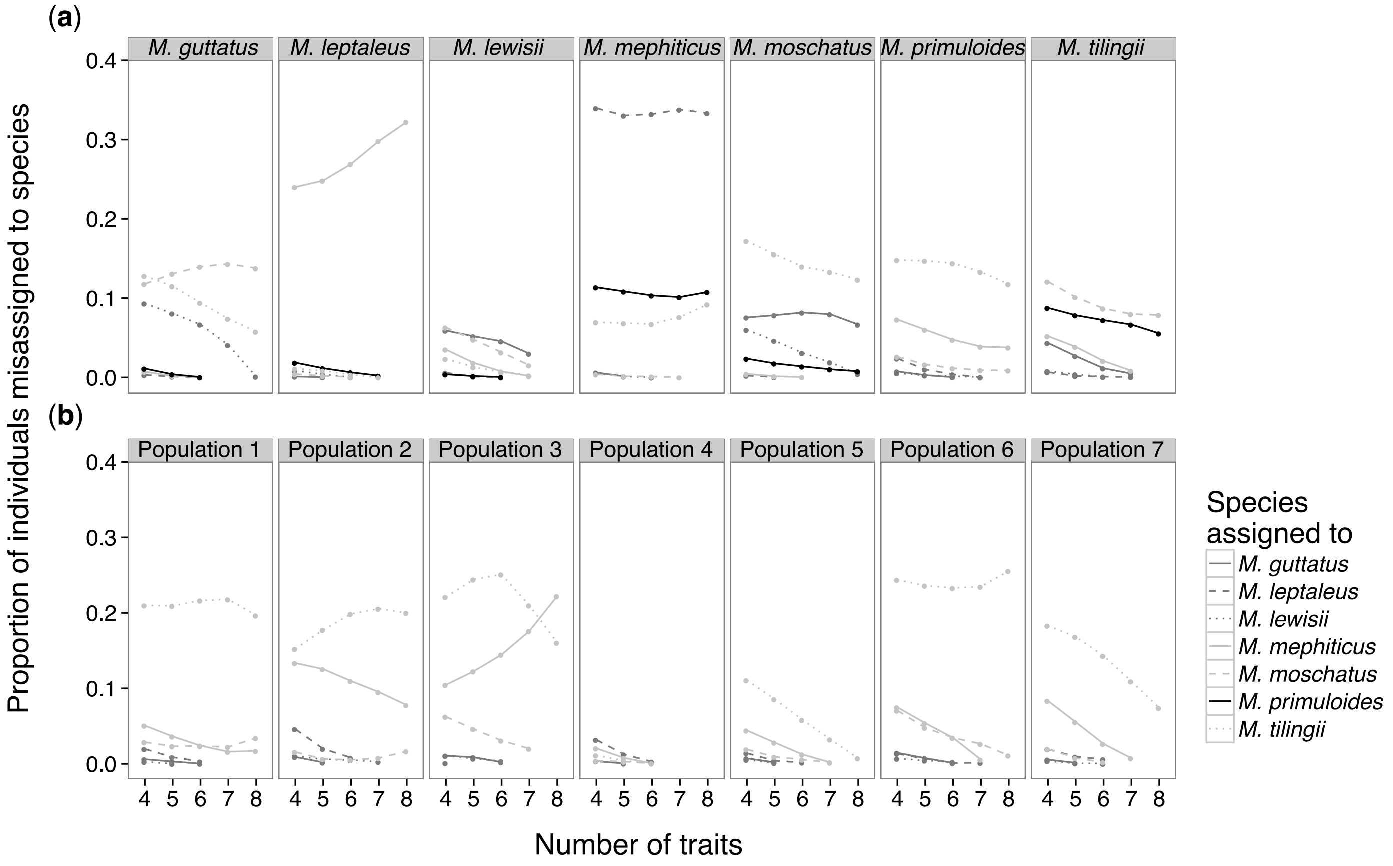

Supplement: Supplementary file 1 [file ECE3-7-2273-s001.pdf]
