# Supplementary material for: Trait dimensionality and population choice alter estimates of phenotypic dissimilarity
Source: Ecol Evol. 2017 Mar 8;7(7):2273–85. doi: 10.1002/ece3.2780 (PMC5383497; doi:10.1002/ece3.2780)
Supplement: Supplementary file 2 [file ECE3-7-2273-s002.docx]

|  | Fl | Buds | Pods | Cor Wid | Cor Len | Herk | Ht | Leaf # | Stem Wid | Int  Node | SLA | Perim | Circ |
| --- | --- | --- | --- | --- | --- | --- | --- | --- | --- | --- | --- | --- | --- |
| Buds | 0.58 |  |  |  |  |  |  |  |  |  |  |  |  |
| Pods | 0.73 | 0.71 |  |  |  |  |  |  |  |  |  |  |  |
| Cor Wid | 0.19 | 0.38 | 0.31 |  |  |  |  |  |  |  |  |  |  |
| Cor Len | -0.09 | 0.05 | 0.03 | 0.32 |  |  |  |  |  |  |  |  |  |
| Herk | 0.05 | 0.36 | 0.26 | 0.25 | 0.21 |  |  |  |  |  |  |  |  |
| Ht | 0.38 | 0.63 | 0.64 | 0.50 | 0.35 | 0.42 |  |  |  |  |  |  |  |
| Leaf # | -0.03 | 0.14 | 0.21 | 0.23 | 0.45 | 0.27 | 0.52 |  |  |  |  |  |  |
| Stem Wid | 0.37 | 0.76 | 0.57 | 0.50 | 0.30 | 0.58 | 0.87 | 0.40 |  |  |  |  |  |
| Int Node | 0.20 | 0.24 | 0.25 | 0.18 | 0.36 | 0.29 | 0.40 | 0.23 | 0.48 |  |  |  |  |
| SLA | -0.16 | -0.15 | -0.10 | -0.05 | 0.48 | 0.20 | -0.01 | 0.15 | -0.01 | 0.32 |  |  |  |
| Perim | 0.17 | 0.50 | 0.38 | 0.41 | 0.33 | 0.58 | 0.71 | 0.48 | 0.79 | 0.30 | 0.01 |  |  |
| Circ | -0.27 | -0.20 | -0.23 | -0.17 | -0.19 | 0.06 | -0.35 | -0.21 | -0.28 | -0.43 | 0.04 | -0.29 |  |
| AR | 0.07 | -0.22 | -0.07 | -0.17 | -0.03 | -0.39 | -0.04 | -0.02 | -0.18 | 0.26 | 0.00 | -0.16 | -0.71 |

**Table S1. Trait correlations.** Pairwise correlations for floral and vegetative traits using all species, populations, and individuals.

Fl = Number of flowers  Ht = Plant height

Buds = Number of flower buds  Leaf # = Number of leaves

Pods = Number of seed pods  Stem Wid = Stem width

Cor Wid = Corolla width   Int Node = Internode (rosette or not)

Herk = Herkogamy (stigma-anther separation) SLA = Specific Leaf Area

Perim = Leaf perimeter

Circ = Leaf circularity

     AR = Leaf aspect ratio (length / width)
