# Supplementary material for: Trait dimensionality and population choice alter estimates of phenotypic dissimilarity
Source: Ecol Evol. 2017 Mar 8;7(7):2273–85. doi: 10.1002/ece3.2780 (PMC5383497; doi:10.1002/ece3.2780)
Supplement: Supplementary file 4 [file ECE3-7-2273-s004.pdf]

Analyses for Carscadden et al. 2016 Trait Dimensionality

Kelly Carscadden

September 11, 2016

NOTE: in some code chunks below, you will have to delete 'eval=F' to actually execute the code if knitting a pdf from the markdown file.

Load packages.

```
require(vegan) #for decostand
require(cluster) #for daisy (Gower's distance)
require(parallel) #for parallel computing
require(pbapply) #for adding a progress bar to apply functions
require(MASS) #for lda function
require(ggplot2) #for sample figure
```

Read in cleaned data.

```
d1<-read.csv("Carscadden2016TraitDimData.csv")

#ensure there are no missing values (for sepPcoa); below test should = F
any(is.na(d1))
```

```
## [1] FALSE
```

```
str(d1)
```

```
## 'data.frame':    181 obs. of  19 variables:
##  $ site          : Factor w/ 12 levels "1.McGurk","10.MayLk",..: 9 9 9 9 9 9 9 9 9 9 ...
##  $ site.num      : num  6 6 6 6 6 6 6 6 6 6 ...
##  $ sp            : Factor w/ 7 levels "gutt","lept",..: 1 1 1 1 1 1 1 1 1 1 ...
##  $ ind           : int  1 2 3 4 5 7 8 9 10 11 ...
##  $ flowers       : int  1 1 1 1 2 1 2 2 6 1 ...
##  $ buds          : int  3 5 4 5 13 4 9 5 13 5 ...
##  $ seed.pods     : int  3 8 1 7 6 1 8 0 20 3 ...
##  $ avg.cor.widest: num  8.4 9.2 9.3 12.7 12.8 9.9 10.8 11.4 11.9 11.1 ...
##  $ avg.cor.len   : num  6.4 7.9 6.2 7.7 6.5 9.3 9.2 8.3 6.8 7.8 ...
##  $ avg.herk      : num  1.8 1.7 1.6 2.6 4 ...
##  $ ht            : num  175 370 225 252 481 ...
##  $ leaf.num      : int  10 14 14 12 26 14 12 14 16 12 ...
##  $ stem.wid      : num  2 3.1 1.9 3.8 6 3.7 3.9 2.8 6.2 3.9 ...
##  $ internode     : int  1 1 1 1 1 1 1 1 1 1 ...
##  $ SLA           : num  27.3 29.6 23.8 31.6 34.6 ...
##  $ avg.perim     : num  64.2 49.1 29.8 69.8 110.1 ...
##  $ avg.circ      : num  0.634 0.536 0.539 0.583 0.515 0.443 0.458 0.58 0.436 0.54 ...
##  $ avg.AR        : num  1.24 1.12 1.13 1.07 1.11 ...
##  $ pop           : Factor w/ 14 levels "gutt_6","lept_7",..: 1 1 1 1 1 1 1 1 1 1 ...
```

Key to column names in the provided dataset:

site: site label

site.num: numeric label of site sampled (numbers not relevant here)

sp: species of Mimulus, abbreviated

```
gutt = Mimulus guttatus

lept = Mimulus leptaleus

lewisii = Mimulus lewisii

meph = Mimulus mephiticus

mosch = Mimulus moschatus

prim = Mimulus primuloides var. primuloides

tilingii = Mimulus tilingii
```

ind: individual sampled

flowers: number of flowers

buds: number of floral buds

seed.pods: number of seed pods

avg.cor.widest: corolla width at the floral aperture, averaged across flowers of an individual

avg.cor.len: corolla length from calyx to aperture, averaged across flowers of an individual

avg.herk: stigma-anther separation (positive numbers indicate stigmas are above anthers)

ht: plant height

leaf.num: number of leaves

stem.wid: basal stem width

internode: whether the plant is a rosette (0) or erect (1)

SLA: specific leaf area

avg.perim: leaf perimeter, averaged across 2 leaves per individual

avg.circ: leaf circularity, averaged across 2 leaves per individual

avg.AR: leaf aspect ratio (length:width), averaged across 2 leaves per individual

pop: population code (combination of sp and site.num)

Standardize trait data.

```
d1.st<-d1

#Need to work with only positive numbers, so add a small amount to
```

```
#all herkogamy values
d1.st$avg.herk<-d1.st$avg.herk +2.9

#standardize to mean=0, variance=1
#the binary variable (internode) is not standardized at this point,
#but Gower's distance performs range-based standardization
d1.st[,c(5:13,15:18)]<-decostand(d1.st[,c(5:13,15:18)],
                                  method="standardize", na.rm=T)
```

Generate a list of all possible vegetative trait combinations, floral trait combinations, and balanced combined (floral/vegetative) trait combinations.

By 'balanced', we mean we only considered those trait combinations featuring roughly equal numbers of vegetative and floral traits.

```
fl<-d1.st[,c(1:10,19)]
veg<-d1.st[,c(1:4,11:19)]

#VEGETATIVE TRAITS
#list of all trait combos, by trait column number
traitcols<-c(5:12)  #refers to column numbers within 'veg' df created above
 for (i in 1:8){    #there are 8 vegetative traits

   #create matrices of combinations of 1:8 vegetative traits
     assign(paste("combo",i,sep=""),combn(traitcols,m=i))
 }

#for example, look at the 8 possible 7-trait combinations in 'combo7',
#indexed by column number
combo7
```

```
##      [,1] [,2] [,3] [,4] [,5] [,6] [,7] [,8]
## [1,]    5    5    5    5    5    5    5    6
## [2,]    6    6    6    6    6    6    7    7
## [3,]    7    7    7    7    7    8    8    8
## [4,]    8    8    8    8    9    9    9    9
## [5,]    9    9    9   10   10   10   10   10
## [6,]   10   10   11   11   11   11   11   11
## [7,]   11   12   12   12   12   12   12   12
```

```
#we'll focus on combinations of 4:8 traits, so store these matrices in a list
vegCombos<-list(combo4,combo5,combo6,combo7,combo8)


#FLORAL TRAITS
traitcols<-c(5:10)
 for (i in 1:6){   #there are 6 floral traits
     assign(paste("combo",i,sep=""),combn(traitcols,m=i))
 }
```

```r
flCombos<-list(combo4,combo5,combo6)


#COMBINED DATASET WITH BOTH VEGETATIVE AND FLORAL TRAITS
vegcol<-c(1:4,11:19)
flcol<-c(1:10,19)

traitcols<-c(5:18) #14 traits
elim<-NULL
k<-0
for (i in 1:14){
    all<-combn(traitcols,m=i) #all possible floral/vegetative trait combos

    #remove combos which DO NOT HAVE ~ balanced numbers of
    #vegetative & floral traits
    for (p in 1:ncol(all)){
            combination<-all[,p]
            numVegTraits<-length(vegcol[vegcol %in% combination])
            numFlTraits<-length(flcol[flcol %in% combination])

        if(numVegTraits < floor(i/2)| numFlTraits < floor(i/2)){
            k<-k+1
            elim[k]<-p #stores the column that needs removing
            elim<-as.vector(na.omit(elim))
        }
    }

#remove the combo that lacks ~even numbers of both vegetative & floral traits
        if(length(elim)>0){all<-all[,-elim]}
        assign(paste("combo",i,sep=""),all)
        elim<-NULL
 }

allCombos_balanced<-list(combo4,combo5,combo6,combo7,combo8,combo9,
                         combo10,combo11,combo12,combo13)
#note, cannot have a balanced combo14 since there are only 6 floral traits
#in our dataset

#create a list of our vegetative, floral, and combined dataset trait
#combination lists
comboList<-list(vegCombos,flCombos,allCombos_balanced)
```

Set up functions and parameters.

```r
#Function to display progress bar for mclapply.
#mclapply is lapply across multiple cores.
pbmclapply<-function (X, FUN, ..., mc.preschedule = TRUE, mc.set.seed = TRUE,
    mc.silent = FALSE, mc.cores = 1L, mc.cleanup = TRUE, mc.allow.recursive = TRUE)
{
    cores <- as.integer(mc.cores)
    if (cores < 1L)
        stop("'mc.cores' must be >= 1")
    if (cores > 1L)
```

```
        stop("'mc.cores' > 1 is not supported on Windows")
    pblapply(X, FUN, ...)
}

#Use an existing function to calculate group means & SE (cookbook-r.com)
summarySE <- function(data=NULL, measurevar, groupvars=NULL, na.rm=FALSE,
                      .drop=TRUE) {
     require(plyr)
     length2 <- function (x, na.rm=FALSE) {
        if (na.rm) sum(!is.na(x))
        else       length(x)
    }

    #For each group, a vector with mean, sd, and n will be generated
    datac <- ddply(data, groupvars, .drop=.drop,
      .fun = function(xx, col) {
        c(N    = length2(xx[[col]], na.rm=na.rm),
          mean = mean   (xx[[col]], na.rm=na.rm),
          sd   = sd     (xx[[col]], na.rm=na.rm)
        )
      },
      measurevar
    )

    #Relabel the 'mean' column
    datac <- rename(datac, c("mean" = measurevar))

    #Calculate SEM
    datac$se <- datac$sd / sqrt(datac$N)

    return(datac)
}


#parameters for analyses
df<-d1.st
nrep<-100 #want 100 reps
minSize<-9 #sample 9 individuals per population
```

Generate balanced species pools

```
#First level of lists will be reps; second level will be unique species
spPools<-pbmclapply(1:nrep, function(j){
  lapply(unique(df$sp), function(m){
         spdat<-df[df$sp==m,]
         poplist<-unique(spdat$pop)
         randpop<-sample(poplist,1,replace=F)

         #data for one randomly selected population
         popdat<-subset(spdat,spdat$pop==randpop)
```

```
        #get balanced data frame by sampling 9 individuals per population
         randEven<-popdat[sample(nrow(popdat),minSize,replace=F),]
         cbind(randEven, run=j)
    }) })

  balanced.samples<-do.call(rbind.data.frame,lapply(spPools, function(j){
          do.call(rbind.data.frame, j)
  }))


#Write to file (optional) and remove from memory,
#since we're not actively calling upon this data frame
write.csv(balanced.samples, "balanced_samples.csv",row.names=FALSE)
rm(balanced.samples)

#Save a list format of the output (i.e., our balanced species pools) instead
save(list="spPools", file="spPools.RDATA")
```

If reading in previously generated species pools:

```
load("spPools.RDATA")
```

With our lists of trait combinations and list of balanced species pools, we're ready to run our analyses. Note, we have not set seed in the above code, so your results may differ slightly from ours due to random sampling.

Let's look at how well individual traits discriminate species.

```
do.call(rbind.data.frame, pbmclapply(1:nrep, function(j){   #Within each rep
    sppvar<-as.data.frame(do.call(rbind,lapply(spPools[[j]], function(m){
         v<-sapply(5:(dim(m)[2]-2), function(x) var(m[,x]))
         names(v)<-names(m)[5:(dim(m)[2]-2)]
         v
     }))) #Calculates variance for each trait, within each species

    #Make contingency table for each trait
    output<-do.call(rbind.data.frame, sapply(1:dim(sppvar)[2], function(T){
         if(all(sppvar[,T]!=0)){
           #Using only traits that are variable within the sub-sampled populations.
           #LDA doesn't work with binary variables, so in this single-trait
           #analysis, the binary internode variable will be dropped.

             dat<-droplevels(as.data.frame(do.call(rbind,spPools[[j]])[,c(19,(T+4))]))
             single.lda<-lda(as.data.frame(dat[,2]),grouping=factor(dat$pop),CV=TRUE)
             single.outcome<-as.data.frame(cbind(pop=as.character(dat$pop),
                                                class=as.character(single.lda$class)))
             single.outcome$class<-factor(single.outcome$class, levels=levels(dat$pop))
             single.tab<-table(single.outcome[,1],single.outcome[,2])
             #How well does lda predict species' ID?

             #Calculate correct assignment probability
```

```
            assnProb<-single.tab/minSize
            assnProbDiag<-diag(assnProb)   #Correct assignment

            do.call(cbind.data.frame,list(pop = rownames(assnProb), rep = j,
                                          Trait = names(spPools[[j]][[1]])[T+4],
                                          assignProb = assnProbDiag,
                                          single.lda$posterior))
        }
    }))
    write.table(output, file="SingleAssnPr.csv", append=TRUE, sep=",",
                col.names=ifelse(j %in% 1,TRUE,FALSE),
                row.names=FALSE);rm(output)
}))
```

This output includes the correct assignment probability (assignProb) for each pop/rep/trait and the posterior (probability of mis-assigning individuals to different species).

To summarize this data:

```
singleAssnPr<-read.csv("SingleAssnPr.csv")

#Remove leaf number and herkogamy, since two populations contain
#estimated values (see main text).
singleAssnPr<-singleAssnPr[singleAssnPr$Trait!="leaf.num",]
singleAssnPr<-singleAssnPr[singleAssnPr$Trait!="avg.herk",]

singleAssnPr$sp<-sapply(strsplit(as.character(singleAssnPr$pop),"\\_"),'[',1)
#Create a column of species names

singleAssnPr.summ<-summarySE(singleAssnPr,measurevar="assignProb",
                             groupvars=c("sp","Trait"))
```

With the above summarized data, you can investigate the average correct assignment using each single trait and explore how this varies across species.

We move on to consider assignment success of trait *combinations.*

First, for each balanced species pool, we subset the main trait dataset to create datasets with different numbers and types of traits.

```
allcol<-c(1:19)
dataframes<-list(vegcol,flcol,allcol)
traitlen<-1:19

#Select a trait dataset for a balanced species pool
#use a)vegetative, b)floral, c)veg/fl combo using separate PCoA axes
#for LDA ("combined constrained"), d)veg/fl combo in a combined PCoA

subdat<-pbmclapply(spPools, function(j){
  #unlist into a dataframe, so the sp chunks of spPools[[j]] are together
    bal<-do.call(rbind.data.frame, j)
```

```
        #For each dataset, pick a trait dataset & number of traits
        #Picking a trait dataset
         subdat<-lapply(1:length(dataframes),function(d){
             remove<-traitlen[!traitlen %in% unlist(dataframes[[d]])]
             #removes cols based on col numbers that don't appear in the desired
             #trait subset df; doesn't subset correctly if d is still a list element

         ifelse(length(remove)>0,subdat<-bal[,-remove],subdat<-bal)
         cbind(subdat,d=d)
         })#end lapply 1:length(dataframes), creating (internal) subdat


         com<-lapply(1:length(dataframes),function(d){
             combos<-comboList[[d]]
             lapply(1:length(combos),function(h){
                 comb<-combos[[h]]

                 #If there is a huge # of trait combos(>100),
                 #take a random subsample of 100 combos
                 colList<-1:ncol(comb)
                 if(ncol(comb)>100){
                     randcol<-sample(colList,100,replace=F)
                     comb<-comb[,randcol]
                 } else{
                     randcol<-colList
                     }#end of if loop needed to generate randcol

                 dat<-lapply(1:ncol(comb), function(i){
                 dat<-subdat[[d]][,c(1:4,(length(subdat[[d]])-2),
                                length(subdat[[d]])-1,length(subdat[[d]]),comb[,i])]

                     rownames(dat)<-c(1:nrow(dat))
                     dat$origCol<-randcol[i]
                     #outputs what the original column was, so can refer to traits later
                     #get data frame with just the correct traits

                     dat$h<-nrow(comb) #IDs number of traits used
                     dat$i<-i #i is an ID variable, in the *last* col
                     dat
                 })
                 })
         })
 }) #end big subdat


#Checking output, using an example:
subdat[[1]][[1]][[2]][[1]]
```

```
##               site site.num      sp ind        pop run d         ht
## 1         6.Wawona      6.0    gutt   7     gutt_6   1 1  1.44914388
## 2         6.Wawona      6.0    gutt   3     gutt_6   1 1  0.66935253
## 3         6.Wawona      6.0    gutt  12     gutt_6   1 1  3.60811846
## 4         6.Wawona      6.0    gutt  15     gutt_6   1 1  2.36278002
```

```
## 5          6.Wawona      6.0     gutt  18      gutt_6   1 1  1.97288435
## 6          6.Wawona      6.0     gutt   4      gutt_6   1 1  0.82647467
## 7          6.Wawona      6.0     gutt   5      gutt_6   1 1  2.15793931
## 8          6.Wawona      6.0     gutt  13      gutt_6   1 1  3.00872659
## 9          6.Wawona      6.0     gutt   9      gutt_6   1 1  1.87977493
## 10         7.TaftPt      7.0     lept   1      lept_7   1 1 -0.27338030
## 11         7.TaftPt      7.0     lept   8      lept_7   1 1 -0.51197318
## 12         7.TaftPt      7.0     lept   2      lept_7   1 1 -0.29665766
## 13         7.TaftPt      7.0     lept   7      lept_7   1 1 -0.55270855
## 14         7.TaftPt      7.0     lept   4      lept_7   1 1 -0.38976707
## 15         7.TaftPt      7.0     lept   5      lept_7   1 1 -0.39558641
## 16         7.TaftPt      7.0     lept   3      lept_7   1 1 -0.27338030
## 17         7.TaftPt      7.0     lept   9      lept_7   1 1 -0.46541847
## 18         7.TaftPt      7.0     lept   6      lept_7   1 1 -0.31411567
## 19          14.WWHL     14.0  lewisii   1  lewisii_14   1 1  0.89048739
## 20          14.WWHL     14.0  lewisii   6  lewisii_14   1 1  1.13489961
## 21          14.WWHL     14.0  lewisii   9  lewisii_14   1 1  2.13000649
## 22          14.WWHL     14.0  lewisii  12  lewisii_14   1 1  2.11836781
## 23          14.WWHL     14.0  lewisii   8  lewisii_14   1 1  1.22218968
## 24          14.WWHL     14.0  lewisii   2  lewisii_14   1 1  1.28038307
## 25          14.WWHL     14.0  lewisii  10  lewisii_14   1 1  2.62465026
## 26          14.WWHL     14.0  lewisii   4  lewisii_14   1 1  1.33857645
## 27          14.WWHL     14.0  lewisii   3  lewisii_14   1 1  0.93704210
## 28         7.TaftPt      7.0     meph   8      meph_7   1 1 -0.38976707
## 29         7.TaftPt      7.0     meph   9      meph_7   1 1 -0.47123781
## 30         7.TaftPt      7.0     meph   1      meph_7   1 1 -0.27919964
## 31         7.TaftPt      7.0     meph   4      meph_7   1 1 -0.37812840
## 32         7.TaftPt      7.0     meph   5      meph_7   1 1 -0.41886377
## 33         7.TaftPt      7.0     meph   3      meph_7   1 1 -0.28501898
## 34         7.TaftPt      7.0     meph   6      meph_7   1 1 -0.42468310
## 35         7.TaftPt      7.0     meph   7      meph_7   1 1 -0.41886377
## 36         7.TaftPt      7.0     meph   2      meph_7   1 1 -0.47705715
## 37         6.Wawona      6.0    mosch   8     mosch_6   1 1  1.44914388
## 38         6.Wawona      6.0    mosch   4     mosch_6   1 1  1.26292505
## 39         6.Wawona      6.0    mosch   1     mosch_6   1 1  0.24454082
## 40         6.Wawona      6.0    mosch   3     mosch_6   1 1  0.04668331
## 41         6.Wawona      6.0    mosch   2     mosch_6   1 1  0.27945685
## 42         6.Wawona      6.0    mosch   9     mosch_6   1 1  0.86139070
## 43         6.Wawona      6.0    mosch  10     mosch_6   1 1  0.23290214
## 44         6.Wawona      6.0    mosch   7     mosch_6   1 1  1.08834490
## 45         6.Wawona      6.0    mosch   6     mosch_6   1 1  1.18727365
## 46 12A.TuolMdwsPond     12.2     prim   3    prim_12.2   1 1 -0.48869583
## 47 12A.TuolMdwsPond     12.2     prim  14    prim_12.2   1 1 -0.62254061
## 48 12A.TuolMdwsPond     12.2     prim   9    prim_12.2   1 1 -0.54688921
## 49 12A.TuolMdwsPond     12.2     prim  12    prim_12.2   1 1 -0.55270855
## 50 12A.TuolMdwsPond     12.2     prim   5    prim_12.2   1 1 -0.50033450
## 51 12A.TuolMdwsPond     12.2     prim  10    prim_12.2   1 1 -0.51197318
## 52 12A.TuolMdwsPond     12.2     prim   2    prim_12.2   1 1 -0.51197318
## 53 12A.TuolMdwsPond     12.2     prim  18    prim_12.2   1 1 -0.55270855
## 54 12A.TuolMdwsPond     12.2     prim  15    prim_12.2   1 1 -0.54688921
## 55     16.HelenLkINF     16.0 tilingii   4 tilingii_16   1 1 -0.36067038
## 56     16.HelenLkINF     16.0 tilingii   3 tilingii_16   1 1 -0.29083832
## 57     16.HelenLkINF     16.0 tilingii   8 tilingii_16   1 1 -0.17445155
## 58     16.HelenLkINF     16.0 tilingii   5 tilingii_16   1 1 -0.22682560
```

```
## 59    16.HelenLkINF     16.0 tilingii   7 tilingii_16   1 1 -0.27338030
## 60    16.HelenLkINF     16.0 tilingii   2 tilingii_16   1 1 -0.20354824
## 61    16.HelenLkINF     16.0 tilingii   6 tilingii_16   1 1 -0.44796046
## 62    16.HelenLkINF     16.0 tilingii   9 tilingii_16   1 1 -0.30247700
## 63    16.HelenLkINF     16.0 tilingii   1 tilingii_16   1 1  0.13397339
##       leaf.num    stem.wid internode          SLA origCol h i
## 1   0.42629103  1.64723384         1 -0.090478759       1 5 1
## 2   0.42629103  0.52789129         1 -0.517196758       1 5 1
## 3   0.42629103  2.85985494         1 -0.962421047       1 5 1
## 4  -0.44066039  2.70439069         1 -0.858201211       1 5 1
## 5   0.42629103  3.88591894         1 -0.776517896       1 5 1
## 6  -0.00718468  1.70941954         1  0.129707983       1 5 1
## 7   3.02714531  3.07750488         1  0.371295971       1 5 1
## 8   0.42629103  4.50777591         1 -0.832746533       1 5 1
## 9   0.42629103  1.08756257         1 -0.609551191       1 5 1
## 10 -0.87413611 -0.34270847         1 -1.243991780       1 5 1
## 11 -0.87413611 -0.46707986         1 -0.814212321       1 5 1
## 12 -0.87413611 -0.28052277         1 -1.086190793       1 5 1
## 13 -1.30761182 -0.52926556         1 -0.871170191       1 5 1
## 14 -0.44066039 -0.40489417         1 -0.380692868       1 5 1
## 15 -0.87413611 -0.46707986         1 -1.167217336       1 5 1
## 16 -0.87413611 -0.34270847         1  1.628430136       1 5 1
## 17 -1.30761182 -0.40489417         1 -1.000306142       1 5 1
## 18 -0.44066039 -0.40489417         1 -0.671946658       1 5 1
## 19  1.72671817  0.27914850         1 -0.112955215       1 5 1
## 20  0.42629103  0.77663408         1 -0.315977312       1 5 1
## 21  1.72671817  1.39849105         1  0.114713273       1 5 1
## 22  0.85976674  0.40351990         1  0.546297330       1 5 1
## 23  1.29324246  0.34133420         1  0.475274858       1 5 1
## 24  1.29324246  0.77663408         1 -0.161499749       1 5 1
## 25  2.16019388  1.27411966         1 -0.262185318       1 5 1
## 26  0.42629103  0.46570559         1  0.164934683       1 5 1
## 27  2.37693174  0.27914850         1  0.562902318       1 5 1
## 28 -0.87413611 -0.34270847         1  0.246912684       1 5 1
## 29 -1.30761182 -0.40489417         1  0.002179982       1 5 1
## 30 -0.87413611 -0.21833708         1 -0.255390155       1 5 1
## 31 -1.30761182 -0.40489417         1 -0.154211463       1 5 1
## 32 -0.87413611 -0.15615138         1 -0.681138756       1 5 1
## 33 -0.44066039 -0.09396568         1 -0.059242447       1 5 1
## 34 -0.87413611 -0.21833708         1  0.016527508       1 5 1
## 35 -0.87413611 -0.40489417         1 -0.050279700       1 5 1
## 36 -1.30761182 -0.46707986         1 -0.522996659       1 5 1
## 37  0.42629103  0.40351990         1  0.989009717       1 5 1
## 38  6.06147529  0.34133420         1  1.112979244       1 5 1
## 39  1.29324246  0.09259141         1  0.302598697       1 5 1
## 40  1.29324246  0.34133420         1  0.421610921       1 5 1
## 41  6.06147529  0.90100547         1 -1.294681572       1 5 1
## 42 -0.00718468  0.52789129         1  0.983406129       1 5 1
## 43  0.85976674  0.27914850         1  1.670867259       1 5 1
## 44  0.85976674  1.14974826         1  0.414471670       1 5 1
## 45  0.85976674  0.03040571         1 -1.359377780       1 5 1
## 46 -0.00718468 -0.65363696         0 -0.694815925       1 5 1
## 47 -1.30761182 -0.65363696         0 -1.291401673       1 5 1
## 48 -0.00718468 -0.65363696         0 -1.044804837       1 5 1
```

```
## 49 -0.44066039 -0.65363696         0 -0.687740770       1 5 1
## 50 -0.00718468 -0.65363696         0 -0.795765848       1 5 1
## 51  0.64302889 -0.65363696         0 -0.456136641       1 5 1
## 52  0.85976674 -0.65363696         0 -0.575216344       1 5 1
## 53 -0.44066039 -0.65363696         0  0.373790316       1 5 1
## 54  0.85976674 -0.65363696         0  0.474285265       1 5 1
## 55 -0.44066039 -0.09396568         1  0.240936503       1 5 1
## 56 -0.00718468  0.03040571         1 -0.281851307       1 5 1
## 57 -0.00718468  0.21696281         1  0.671596388       1 5 1
## 58 -0.00718468  0.15477711         1 -0.507665502       1 5 1
## 59 -0.00718468  0.03040571         1 -0.019157303       1 5 1
## 60  0.20955318  0.09259141         1 -0.528511681       1 5 1
## 61 -0.44066039  0.34133420         1  1.809061051       1 5 1
## 62 -0.44066039  0.15477711         1  0.014853137       1 5 1
## 63  0.42629103  0.15477711         1 -0.491985354       1 5 1
```

```
#shows rep 1 (run=1), dataset 1 (d=1), 5 traits used (h=5),
#trait combo selected (i=1)

save(list="subdat", file="subdat.RDATA")
```

If reading in previously generated subsetted data:

```
load("subdat.RDATA")
```

With these trait datasets, perform a PCoA, extract 2 major axes, and input them into an LDA.

```
vegtraits<-names(veg)[5:12]
fltraits<-names(fl)[5:10]
alltraits<-names(df)[5:18]


#Create distance matrix
pbmclapply(subdat, function(n){ #n is the number of datasets within each rep
    lapply(n, function(d){  # d is the different numbers of traits
        lapply(d, function(h){  #h is for each of the 70 combos (i)
            lapply(h, function(i){  #i is each specific combo.
              #i is the lowest level which produced dat.

            #create a distance matrix
            traitlist<-names(i[,8:(length(i)-2)])
            ifelse(any(traitlist=="internode"),
                   gow.dis<-daisy(i[,8:(ncol(i)-3)],"gower",stand=F,
                                  type=list(symm="internode")),
                   gow.dis<-daisy(i[,8:(ncol(i)-3)],"gower",stand=F))
                   #range-based Gower standardization is applied
                   #two different options depending on if binary variable (internode)
                   #is present or not

            #Perform PCoA to generate composite 'trait' axes for LDA
            pcoa.gow<-cmdscale(gow.dis,eig=T)

            #If you want to look at trait loadings and other aspects of the
```

```
      #PCoA output, store those here.
#We have removed it for speed and simplicity.
      #To be able to compare trait loadings, you will need to flip
  #all Axis 1 so they are positively correlated (and similarly with Axis 2).

  ###LDA using PCoA axes as input

      #Removing levels of sp,pop factor, so just applies to data present and
      #not full subdat options
      i2<-droplevels(i)

      ldClass<-lda(i2$pop~pcoa.gow$points[,1:2],CV=TRUE)$class
      outcome<-as.data.frame(cbind(pop=as.character(i2$pop),
                   class=as.character(ldClass)))
      outcome$class<-factor(outcome$class, levels=levels(i2$pop))
      #keeps all pops, even if 0 ind were assigned to a particular pop class

      tab<-table(outcome[,1],outcome[,2]) #how well does lda predict pop ID?

      #Calculate correct assignment probability
      assnProb<-tab/minSize
      assnProbDiag<-diag(assnProb)    #correct assignment

      ldaAssnOutput<-do.call(cbind.data.frame,list(pop = rownames(assnProb),
                    run = i$run[1], d=i$d[1], dim=i$h[1],
                    i=i$i[1],origCol=i$origCol[1], method="NA",
                    assignProb = assnProbDiag))


      #So we can calculate mis-assignment of individuals to species later,
      #save class
      classOutput<-do.call(cbind.data.frame,list(pop=i2$pop,run = i$run[1],
                     d=i$d[1], dim=i$h[1], i=i$i[1],origCol=i$origCol[1],
                     method="NA",class=ldClass))

  write.table(classOutput, file="classOutput.csv", append=TRUE,
            sep=",", row.names=FALSE,
            col.names=ifelse(i  $run[1] %in% 1 && i$d[1]==1 && i$h[1]==4
            && i$i[1]==1,TRUE,FALSE))
  rm(classOutput)


  write.table(ldaAssnOutput, file="ldaAssnOutput.csv", append=TRUE,
            sep=",", col.names=ifelse(i$run[1] %in% 1 && i$d[1]==1 &&
            i$h[1]==4 && i$i[1]==1,TRUE,FALSE),row.names=FALSE)
  rm(ldaAssnOutput)

  ##############
  #For vegetative-floral trait combinations, we also want to perform
  #another ordination, constrained such that
#one pcoa axis is vegetative and one is floral ("combined constrained")

  if(i$d[1]==3){ #split traits into veg and fl
```

```
    traitlist2<-names(i[,8:(length(i)-3)]) #what traits have been picked
    veg3<-vegtraits[vegtraits %in% traitlist2]
    fl3<-fltraits[fltraits %in% traitlist2]

    #subset the data accordingly
    vegdat<-as.data.frame(i[,veg3]);colnames(vegdat)<-c(veg3)
    #as.data.frame so it can deal with one-trait situations

    fldat<-as.data.frame(i[,fl3]);colnames(fldat)<-c(fl3)

    gow.dis.veg<-NULL

  ifelse(any(veg3=="internode"),
         gow.dis.veg<-daisy(vegdat,"gower",stand=F,
                            type=list(symm="internode")),
         gow.dis.veg<-daisy(vegdat,"gower",stand=F))

  gow.dis.fl<-NULL
  gow.dis.fl<-daisy(fldat,"gower",stand=F)

#running PCoA on fl and veg separately
pcoa.gow.veg<-cmdscale(gow.dis.veg,eig=T)
pcoa.gow.fl<-cmdscale(gow.dis.fl,eig=T)

#if you want to look at trait loadings, other PCoA output,
#store those here.
#We have removed it for speed and simplicity.
#To be able to compare trait loadings, you will need to flip
  #all Axis 1 so they are positively correlated (and similarly with Axis 2).

  #pulling first axis of each separate PCoA ("sepPcoa" in the below code)
#to feed into LDA

    ldClass.sep<-lda(i2$pop~pcoa.gow.veg$points[,1]+
                       pcoa.gow.fl$points[,1],CV=TRUE)$class
    outcome.sep<-as.data.frame(cbind(pop=as.character(i2$pop),
                       class=as.character(ldClass.sep)))
    outcome.sep$class<-factor(outcome.sep$class, levels=levels(i2$pop))
    tab.sep<-table(outcome.sep[,1],outcome.sep[,2])
    #how well does lda predict pop ID?

    #Calculate correct assignment probability
    assnProb.sep<-tab.sep/minSize
    assnProbDiag.sep<-diag(assnProb.sep)    #correct assignment

    ldaAssnOutput.sep<-do.call(cbind.data.frame,
                        list(pop = rownames(assnProb.sep), run = i$run[1],
                        d=i$d[1], dim=i$h[1],
                        i=i$i[1],origCol=i$origCol[1], method="sepPcoa",
                        assignProb = assnProbDiag.sep))

write.table(ldaAssnOutput.sep, file="ldaAssnOutput.csv", append=TRUE,
                    sep=",", col.names=FALSE, row.names=FALSE)
```

```
        rm(ldaAssnOutput.sep)

          #So we can calculate mis-assignment of individuals to species later,
        #save class
            classOutput.sep<-do.call(cbind.data.frame,
                                list(pop=i2$pop,run = i$run[1], d=i$d[1],
                                dim=i$h[1], i=i$i[1],origCol=i$origCol[1],
                                method="sepPcoa",class=ldClass.sep))

          write.table(classOutput.sep, file="classOutput.csv", append=TRUE,
                              sep=",", row.names=FALSE, col.names=FALSE)
          rm(classOutput.sep)

    }#end of sepPcoa set

            })
        })
    })
})  #end pbmclapply, ldaAssnOutput
```

Now we can summarize the data, like we did above.

First, how did trait datasets differ in assignment success?

```
assign.outcome.gow<-read.csv("ldaAssnOutput.csv",header=T, stringsAsFactors=F)
str(assign.outcome.gow)
```

```
## 'data.frame':    1299900 obs. of  8 variables:
##  $ pop       : chr  "gutt_6" "lept_7" "lewisii_14" "meph_7" ...
##  $ run       : int  1 1 1 1 1 1 1 1 1 1 ...
##  $ d         : int  1 1 1 1 1 1 1 1 1 1 ...
##  $ dim       : int  4 4 4 4 4 4 4 4 4 4 ...
##  $ i         : int  1 1 1 1 1 1 1 2 2 2 ...
##  $ origCol   : int  1 1 1 1 1 1 1 2 2 2 ...
##  $ method    : chr  NA NA NA NA ...
##  $ assignProb: num  0.667 0.444 0.667 0.333 0.444 ...
```

```
assign.outcome.gow$assignProb<-as.numeric(assign.outcome.gow$assignProb)

#create an identifier based on trait dataset and whether vegetative-floral
#combinations used a single PCoA or a constrained (2 separate PCoAs) approach
assign.outcome.gow$approach<-paste(assign.outcome.gow$d,
                          assign.outcome.gow$method,sep=".")
```

Key to 'Approach' identifier:

1.NA: Vegetative (single PCoA for all selected vegetative traits)

2.NA: Floral (single PCoA for all selected floral traits)

3.NA: Combined (single PCoA for all selected vegetative & floral traits)

3.sepPcoa: Combined (constrained - one PCoA of only vegetative traits and one PCoA of only floral traits. The first axis of each was taken as input for the LDA)

```r
#summarize data
dfc<-summarySE(assign.outcome.gow,measurevar="assignProb",
                 groupvars=c("approach","dim"))
                 #in dfc, assignProb is now a mean

dfc$dim<-as.numeric(dfc$dim)

#visualize trends given the randomly sampled species pools and trait
#combinations. This figure code is included as an example
#lines fit for illustration
ggplot(dfc,aes(x=factor(dim),y=assignProb,col=approach))+
geom_point()+
geom_smooth(aes(group=approach),se=F)+
geom_errorbar(aes(ymin=assignProb-se, ymax=assignProb+se),width=0.25)+
xlab("Number of traits")+
ylab("Mean proportion of individuals\ncorrectly assigned to species")+
theme(text=element_text(family="Helvetica"))+
scale_color_manual(values=c("gray81","gray44","gray0","gray60"),
                   name="Trait Dataset",labels=c("Vegetative", "Floral",
                   "Combined","Combined constrained"))+
theme_bw()+
theme(panel.grid.major = element_blank())+
theme(panel.grid.minor=element_blank())+
theme(axis.text.x=element_text(size=14))+
theme(axis.text.y=element_text(size=14))+
theme(axis.title.y=element_text(size=18))+
theme(axis.title.y = element_text(hjust = .4))+
theme(axis.title.x=element_text(size=18))+
theme(axis.title.x = element_text(vjust = .4))+
theme(legend.title = element_text(size=18,face="plain"))+
theme(legend.text = element_text(size=14))+
theme(legend.position=c(0.78,0.15)) #requires relative coords btwn 0 and 1
```

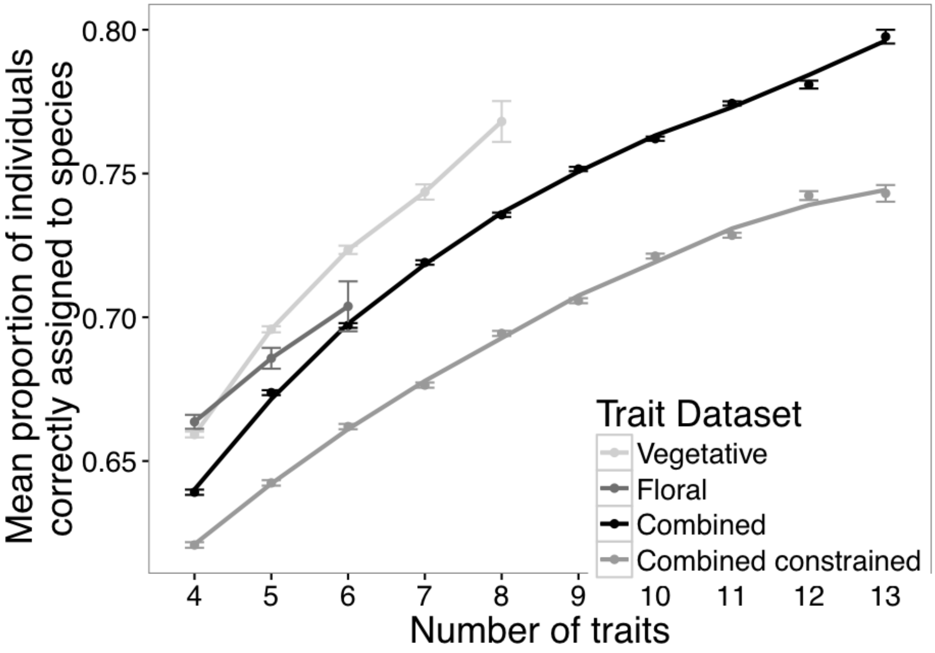

Next, see if the number of trait groups included in a trait combination influence assignment success. Simultaneously calculate the average pairwise correlation coefficient within each trait combination.

```
assign.outcome.gow$dim<-as.numeric(assign.outcome.gow$dim) #to index
assign.outcome.gow$i<-as.numeric(assign.outcome.gow$i)

#for vegetative dataset (d=1)
assign.outcome.veg<-assign.outcome.gow[assign.outcome.gow$d==1,]
assn.i.veg<-summarySE(assign.outcome.veg,measurevar="assignProb",
                      groupvars=c("dim","i"))
#for the points to overlay on boxplot
#(points indicate average success of each trait combo)

#for floral dataset (d=2)
assign.outcome.fl<-assign.outcome.gow[assign.outcome.gow$d==2,]
assn.i.fl<-summarySE(assign.outcome.fl,measurevar="assignProb",
                     groupvars=c("dim","i"))

#determine pairwise correlation between traits
correl<-cor(d1.st[,5:18])
upper<-correl
upper[upper.tri(correl,diag=T)]<-"" #hide upper triangle,
                                    #diag removes the 1 correl along diag
correldf<-as.data.frame(upper)
write.csv(correldf,file="traitCorrelDf")

#determine whether the number of trait groups included in a combination
```

```r
#influence assignment success
combo<-comboList[[1]]  #vegCombos
assn.i.trait.veg<-NULL

for(h in unique(assn.i.veg$dim)){ #h is 4-8 here
     subh<-assn.i.veg[assn.i.veg$dim==h,]
     comb<-as.data.frame(combo[h-3]) #select the right ref combo

     for(m in unique(subh$i)){
         subm<-subh[subh$i==m,]
         ifelse(any(comb[m]==5|comb[m]==6),size<-1,size<-0)
         ifelse(any(comb[m]==7|comb[m]==8),growthform<-1,growthform<-0)
         ifelse(any(comb[m]==9|comb[m]==10|comb[m]==11|comb[m]==12),
                              leaf<-1,leaf<-0)


         #find avg absolute correlation btwn selected traits
         #select all the relevant rows and cols from the cor matrix
         com<-as.vector(unlist(comb[m]))
         avg.cor<-mean(abs(as.numeric(upper[c(com-4),c(com-4)])),na.rm=T)


         assn.tr<-data.frame(subm,size,growthform,leaf,avg.cor)
         assn.tr$axes<-rowSums(assn.tr[c("size","growthform","leaf")])
         #sum=3 means has a trait from all 3 vegetative trait groups:
         #(size, leaf, growth form)
         assn.i.trait.veg<-rbind(assn.i.trait.veg,assn.tr)
         }
     }

head(assn.i.trait.veg);unique(assn.i.trait.veg$axes)
```

```
##   dim i   N assignProb        sd          se size growthform leaf
## 1   4 1 700  0.5623810 0.2523791 0.009539033    1          1    0
## 2   4 2 700  0.6468254 0.2051406 0.007753586    1          1    1
## 3   4 3 700  0.5700000 0.2117702 0.008004160    1          1    1
## 4   4 4 700  0.6885714 0.1884529 0.007122848    1          1    1
## 5   4 5 700  0.7458730 0.2457460 0.009288327    1          1    1
## 6   4 6 700  0.6592063 0.1768017 0.006682476    1          1    1
##     avg.cor axes
## 1 0.4812759    2
## 2 0.3644733    3
## 3 0.4485151    3
## 4 0.6103787    3
## 5 0.3975869    3
## 6 0.2686652    3

## [1] 2 3 1
```

```r
#Repeat the same procedure for floral traits
 combo<-comboList[[2]] #flCombos

 assn.i.trait.fl<-NULL
```

```
for(h in unique(assn.i.fl$dim)){ #dim is 4-6 here
    subh<-assn.i.fl[assn.i.fl$dim==h,]
    comb<-as.data.frame(combo[h-3]) #select the right ref combo

    for(m in unique(subh$i)){
        subm<-subh[subh$i==m,]
        ifelse(any(comb[m]==5|comb[m]==6|comb[m]==7),str<-1,str<-0)
        ifelse(any(comb[m]==8|comb[m]==9),flsize<-1,flsize<-0)
        ifelse(any(comb[m]==10),herk<-1,herk<-0)

        #find avg absolute correlation btwn selected traits
        #select all the relevant rows and cols from the cor matrix
        com<-as.vector(unlist(comb[m]))
        avg.cor<-mean(abs(as.numeric(upper[c(com-4),c(com-4)])),na.rm=T)


        assn.tr<-data.frame(subm,str,flsize,herk,avg.cor)
        assn.tr$axes<-rowSums(assn.tr[c("str","flsize","herk")])
        #sum=3 means has a trait from all 3 floral trait groups
        assn.i.trait.fl<-rbind(assn.i.trait.fl,assn.tr)
        }
}

head(assn.i.trait.fl); unique(assn.i.trait.fl$axes)
```

```
##   dim i   N assignProb        sd          se str flsize herk   avg.cor
## 1   4 1 700  0.6606349 0.2256115 0.008527312   1      1    0 0.4812759
## 2   4 2 700  0.5569841 0.2894277 0.010939341   1      1    0 0.3644733
## 3   4 3 700  0.4311111 0.2822927 0.010669660   1      0    1 0.4485151
## 4   4 4 700  0.7058730 0.2075869 0.007846048   1      1    0 0.2686652
## 5   4 5 700  0.6782540 0.2105245 0.007957080   1      1    1 0.3017302
## 6   4 6 700  0.6569841 0.2147728 0.008117648   1      1    1 0.2243512
##   axes
## 1    2
## 2    2
## 3    2
## 4    2
## 5    3
## 6    3

## [1] 2 3
```

```
#We then used summarySE to summarize by 'dim' and 'axes'
```

Relate the average success of each combination to the average pairwise correlation coefficient within each trait combination.

```
coef(lm(assignProb ~ avg.cor, data = assn.i.trait.veg))
```

```
## (Intercept)     avg.cor
##   0.7331474  -0.1370863
```

```r
coef(lm(assignProb ~ avg.cor, data = assn.i.trait.fl))
```

```
## (Intercept)     avg.cor
##   0.8411038  -0.5627479
```

Summarize data to see how assignment success varies with the number of traits included, for species and populations.

```r
#for species portion of Fig. 7
assign.outcome.gow$sp<-sapply(strsplit
                         (as.character(assign.outcome.gow$pop),"\\_"),'[',1)
dfc2<-summarySE(assign.outcome.gow,measurevar="assignProb",
                        groupvars=c("approach","sp","dim"))

#for (M. primuloides) population portion of Fig. 7
dfc3<-summarySE(assign.outcome.gow,measurevar="assignProb",
                        groupvars=c("approach","pop","dim"))
dfc3$dim<-as.numeric(dfc3$dim)
dfc3$sp<-sapply(strsplit(as.character(dfc3$pop),"\\_"),'[',1)
dfc.prim<-dfc3[dfc3$sp=="prim",]
```
